# Correlates of screen time in the early years (0–5 years): A systematic review

**DOI:** 10.1016/j.pmedr.2023.102214

**Published:** 2023-04-19

**Authors:** S.L.C. Veldman, T.M. Altenburg, M.J.M. Chinapaw, J.S. Gubbels

**Affiliations:** a Department of Public and Occupational Health, Amsterdam UMC, Vrije Universiteit Amsterdam, Amsterdam Public Health Research Institute, Amsterdam, The Netherlands; b Mulier Institute, Utrecht, The Netherlands; c Department of Health Promotion, NUTRIM School of Nutrition and Translational Research in Metabolism, Maastricht University, Maastricht, The Netherlands

**Keywords:** Screen time, Early childhood, Infants, Toddlers, Pre-schooler, Sedentary behaviour, Correlates, Review

## Abstract

The majority of young children engage in high levels of screen time. To inform future interventions, knowledge on correlates of screen time is important. This review expands on previous work by focusing on the entire early childhood range, and including a broad focus regarding types of correlates and screens. A literature search (PubMed, Embase, PsycINFO, SPORTDiscus) was performed from 2000 up to October 2021. Included studies (cross-sectional and prospective) examined associations between a potential correlate and screen time (duration or frequency) in typically developing, apparently healthy children aged 0–5 years. Methodological quality was assessed by two independent researchers. Fifty-two of 6,614 studies were included. Two studies had high methodological quality. We found moderate evidence for a positive association between an electronic device in the bedroom, parental screen time, having a TV on at home, descriptive norms and screen time, and a negative association between sleep duration, household features, high value on physical activity, monitoring screen time, being in childcare, parental self-efficacy and screen time. We found no evidence for an association for child sex, body mass index, physical activity, temperament, number of siblings, being a first-born, neighborhood-related factors, socio-economic indicators, and parental marital status, physical activity, weight status, depression, wellbeing, sex, age and positive outcome expectations. The evidence for other investigated correlates was inconsistent or insufficient. Despite the evidence for moderate associations, we were unable to draw strong conclusions. More high-quality research is needed to identify correlates of screen time in early childhood.

## Highlights

• This review included 53 studies examining 91 correlates of screen time.
• Studies should focus on sociocultural, environmental, behavioral and economic factors.
• Improved methodology is recommended to ensure high-quality studies.
• Interventions should focus on physical environment and sociocultural factors.

## 1 Introduction

Screens (e.g., television (TV) or mobile phone), are nowadays omnipresent. This has led to substantial increases in screen time in early childhood (children aged < 5 years) (Barber, 2017, Downing et al., 2015, Vanderloo, 2014, McNeill, 2019). The World Health Organization (WHO) screen time guidelines recommend no screen time for infants and toddlers up to age 2 years (World Health Organization, 2019), and<1 h/day for children aged 2–4 years (World Health Organization, 2019). Currently, many young children are exceeding these screen time recommendations (Barber, 2017, Downing et al., 2015, Vanderloo, 2014, McNeill, 2019). A review from 2015 estimated 2.3% to 83% of children aged < 2 years are meeting the screen time recommendation (Downing et al., 2015). More recent studies estimate that 25% of children aged 12 months from the UK meet the guidelines (Barber, 2017), and that Australian preschoolers spent 2.4 h/day on electronic media (McNeill, 2019).

Given the high levels of screen time in young children, there is a growing concern about the impact of screen time on their health and development. Evidence suggest that higher levels of screen time in young children are associated with higher levels of adiposity, more sleep problems and lower scores on measures of psychosocial health, cognitive development and motor development (McNeill, 2019, Li, 2020, Poitras, 2017), highlighting the need to intervene at an early age.

Interventions aimed at reducing screen time in early childhood are effective (Downing, 2018), however, heterogeneity of studies (e.g., intervention setting) and a lack of clear trends in subgroup analyses (e.g., different age groups) have resulted in a need for more research. Examining correlates of screen time is an important step towards identifying effective strategies to reduce screen time. This is especially important as sedentary behavior, including screen time, tracks from early childhood to adolescence and into adulthood (Jones, 2013, Busschaert, 2015). A previous review on correlates of sedentary behaviors in preschoolers concluded that sex and outdoor play were not associated with time spent TV viewing (Hinkley, 2010). A review specifically examining the childcare setting revealed that higher staff education was associated with less screen-viewing activities in preschoolers while home-based care (compared to center-based childcare) was associated with more screen-viewing activities (Vanderloo, 2014). A review examining correlates in children aged < 7 years found several socio-demographic (e.g., higher child age, low socioeconomic position households, non-white ethnicities), sociocultural (e.g., living in higher TV-viewing households less parental screen-viewing rules, maternal depressive symptoms, higher parental body mass index) and environmental (e.g., greater access to media sources and less perceived safety in neighborhoods) correlates of higher screen time (Hoyos Cillero and Jago, 2010). Both reviews included all types of screen time as a total outcome measure. A review examining parental influences in children aged < 6 years concluded that parental screen time was positively associated with young children’s screen time (Xu et al., 2015). Finally, a review in children aged < 3 years concluded that an older age, a minority race/ethnicity, a higher body mass index (BMI), maternal distress/depression, and maternal TV viewing time were associated with higher screen time (Duch, 2013). Additionally, cognitive stimulation in the home environment was associated with lower screen time (Duch, 2013).

Previous reviews have examined specific contexts (Vanderloo, 2014, Xu et al., 2015), age groups (Vanderloo, 2014, Hinkley, 2010, Duch, 2013), mostly or only examined TV viewing as type of screen time (Hinkley, 2010, Duch, 2013) and were all conducted several years ago (<2015). Therefore, and due to the continuous development of screen-based devices (e.g., smartphones) an updated overview of the correlates of (different types of) screen time in early childhood is required. This review expands on previous work by focusing on the entire range of early childhood (0–5 years), and including a broad focus regarding types of correlates and types of screen time. The aim of this systematic review is to identify correlates of screen time in typically developing children aged 0–5 years. Variables reported as potential correlates of screen time were categorized using an ecological perspective (Stokols, 1992, Sallis et al., 2008, Swinburn et al., 1999).

## 2 Methods

### 2.1 Protocol and registration

A review protocol was developed based on the Preferred Reporting Items for Systematic Reviews and Meta-Analyses (PRISMA)-statement (Moher, 2009). This systematic review was registered with the International Prospective Register of Systematic Reviews (PROSPERO: CRD42020204330).

### 2.2 Eligibility criteria

Studies were included if they 1) examined the association between a potential correlate and any type of screen time (total screen time as well as specific types of screen time) in typically developing, apparently healthy children aged 0–5 years (operationalized as a mean age of < 5 years at the time of screen time assessment), 2) used a longitudinal or cross-sectional design, 3) assessed screen time quantitatively (duration and/or frequency) and 4) were written in English and published after 2000, in a peer reviewed scientific journal.

Exclusion criteria were: studies 1) solely presenting data on children born preterm (<37 weeks), 2) using adherence to guidelines (yes/no) as sole assessment of screen time, 3) examining a prenatal potential correlate, except for prenatally assessed socio-demographic factors as these contribute to identifying which groups should be targeted for interventions. The review was conducted with a view to informing interventions to limit screen time.

### 2.3 Literature search and study selection

A comprehensive search from inception up to 26 October 2021was performed in the bibliographic databases PubMed, Embase, PsycINFO and SPORTDiscus. The following terms were used (including synonyms and closely related words) as index terms or free-text words: “Infant”, “Screen time”, “correlate*”. The search was performed without language or publication status restriction. The full search strategies for all databases can be found in supplementary File 1.

One reviewer (SLCV) individually screened all titles and abstracts after removal of duplicates, and a second (JSG) and third reviewer (TMA) each individually and independently screened 50%. Following screening, discrepancies were discussed until consensus was reached. Full text articles were screened by one reviewer (SLCV). Ten percent of the inclusion and each of the exclusion categories were checked by a second reviewer (JSG). Discrepancies (<5%) were discussed until consensus was reached.

### 2.4 Data extraction

Data on the following variables were extracted using a structured form: study methodology (e.g. design, study duration and points of data collection), participants (e.g. sample size, mean age, percentage girls), screen time (e.g. outcome variable, assessment tool used), correlate (e.g. type, assessment tool used) and results. Statistical significance of *p* < 0.05 was accepted as significant. Data extraction was individually conducted by two researchers per article (SLCV for all articles, JSG for 75% of articles and LV for 25% of articles). Results were compared and discrepancies were discussed until consensus was reached.

Variables reported as potential correlates of screen time were categorized. in seven categories that were divided into two main categories, using an ecological perspective (Stokols, 1992, Sallis et al., 2008, Swinburn et al., 1999): 1) Individual (including subcategories Biological, Behavioral attributes and skills, and Cognitive, emotional or psychological); and 2) Environmental (including subcategories Physical, Economic, Political, and Sociocultural).

### 2.5 Quality assessment

Methodological quality of all included studies was determined using an adjusted version of the ‘Quality Assessment Tool for Quantitative Studies’ (EPHPP) (Jackson and Waters, 2005, Thomas, 2004) (see supplementary file 2). Two reviewers each independently scored all included studies (SLCV and either TMA or MJMC). The EPHPP tool contains 19 items divided over eight quality criteria: selection bias, study design, confounders, blinding, data collection methods, withdrawals and drop-outs, intervention integrity, data-analysis. Per quality criterion, a quality score was provided: good, fair or poor. Discrepancies were discussed until consensus was reached. The overall methodological quality of a study was classified as ‘high’ when none of the quality criteria were scored as poor. A study was classified as ‘moderate’ when at most one quality criterion was scored as poor. The overall methodological quality of a study was classified as ‘weak’ when two or more quality criteria were scored as poor. If a study was the only one examining a particular correlate, the methodological quality was not assessed as a best evidence synthesis was not possible (see *Synthesis of evidence).*

### 2.6 Synthesis of evidence

A best evidence synthesis was applied to draw conclusions on the level of evidence for the association between a potential correlate and screen time in children aged 0–5 years. This synthesis was based on the number of studies, their methodological quality and the consistency of findings (Slavin, 1995):

• Strong evidence: consistent findings in multiple studies (≥2) of high methodological quality.
• Moderate evidence: consistent findings in one study of high methodological quality and at least one study of weak or moderate methodological quality, or consistent findings in multiple studies (≥2) of weak or moderate methodological quality.
• No evidence: consistent findings for no significant association in multiple studies (≥2) of moderate or high methodological quality.
• Inconsistent evidence: inconsistent findings in multiple studies (≥2).
• Insufficient evidence: only one study available.

Findings were considered consistent when ≥ 75% of studies demonstrated findings in the same direction, based on significance of *p <* 0.05 in the fully adjusted model. If two or more studies of high methodological quality were available, results of studies with weak methodological quality were ignored in determining level of evidence.

As there was a large variation in how correlates were defined and measured, we combined studies examining a similar construct. Additionally, if studies examined multiple associations reflecting associations with multiple measures of one potential correlate (e.g. house features: presence of a yard, fences or covered outdoor area), they were considered to add evidence when consistently demonstrating a significant association in > 50% of examined associations.

For type of screen use, we aimed to examine the correlates per type of screen time. However, given the large number of correlates for which we had to conclude insufficient or inconsistent evidence based on two studies, we decided to combine all types of screen time to examine correlates. If studies examined multiple associations for different types of screen time (e.g. analyzing the association with multiple assessments of screen time such as TV viewing, playing video games and computer use), they were considered to add evidence when consistently demonstrating a significant association in > 50% of examined associations. Besides total screen time, we also examined TV time (see summary tables in supplementary file 3).

## 3 Results

### 3.1 Overview of studies

**Fig. 1:**
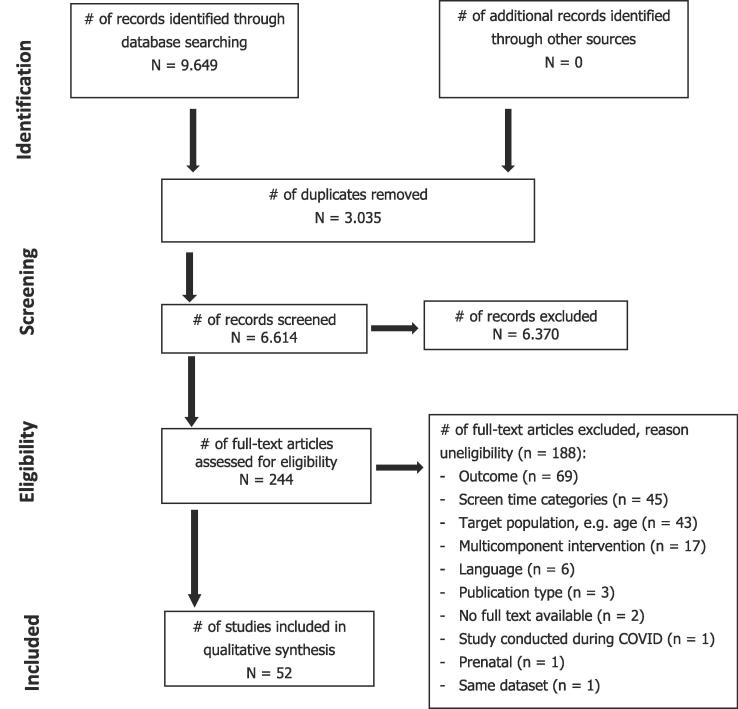
PRISMA Flowchart.

The search identified 6,618 hits after removal of duplicates. In total, 53 studies were included in this review. Fig. 1 presents the flow diagram. Table 1 presents the characteristics of included studies and the results on associations with screen time. Supplementary file 4 presents the methodological quality of included studies.

**Table 1:** Characteristics of included studies and results regarding correlates of screen time, sorted by study design, analysis and first author.

**Reference (Author (year), country)**	**Sample size (n included in analysis)**	**age at (first) screen time assessment, %girls at baseline**	**Screen time assessment, follow-up assessments screen time**	**Correlate(s) examined, association (+/0/-)^b^**
**Observational studies, longitudinal analysis**				
Barber et al. (2017), UK	1558	6.7 ± 0.7mo, 51%	Q: TV time (hrs/day) at ages 6, 12, 18, 24, and 36mo.	Ethnicity (+for Pakistani vs White-British), first-born child (+), maternal TV time (+), more TV on in the house at 24mo (+), maternal attitude at 24mo (+for not agreeing it is importance too not watch much TV vs agreeing), maternal psychological distress at 12 and 24mo (0)
Hish et al. (2021), USA	235	24mo, 45%	Q: TV time (mins/day) at age 2yrs.	Sex (+for girls), race/ethnicity (+for non-Latino black vs Latino Black), parental education (0), income (0), language (0), number of adults in the house (0), number of children in household (- for each additional child), away from home care (-), TV in room where child sleeps (+), earlier age of TV introduction (+)
Hnatiuk et al. (2015), Australia	404	3.8 ± 1.3mo, 46%	Q: TV time (mins/wk) at ages 4 and 19mo.	Maternal self-efficacy for limiting TV viewing from 4 to 19mo (- for high/increasing self-efficacy vs low self-efficacy)
Krogh et al. (2021), Denmark	1095–1580	2.1 ± 0.1mo, 49%	Q: screen time (mins/day) at ages 2, 4, 7 and 11mo.	High maternal educational level at 2mo (-), maternal age at birth (0), household income at 2mo (0)
Matarma et al. (2016), Finland	634	13mo, 48%	Q: screen time (min/day) at ages 0, 13, 24 and 36mo.	Sex (0), lower BMI child at 13mo (-), family income (0), first-born child (0), maternal BMI at baseline (0), higher maternal age (+), higher maternal education (-), full time work mother (+), >4hrs parental sitting time at 13mo (- for fathers; 0 for mothers), >2hrs parental screen time at 13mo (+for mothers; 0 for fathers), not in day care (+),
Morrissey et al. (2014), USA	4750–10700	10.5mo, 49%	Q: screen time (hrs/day) at ages 9mo, 2 and ∼ 4yrs.	Maternal depressive symptoms score (+), Mother has moderate/severe depressive symptoms (+), Duration of maternal depressive symptoms (+)
Thompson et al. (2015), USA	835	±3.5yrs, 50%	I: TV time (hrs/day) assesses twice, every 16mo.	Maternal TV content regulation at 2.1 ± 1.4yrs (-)
Xu et al. (2016), Australia	369–497	1 yr, 50%	I: screen time (hrs/day) at ages 1, 2, 3.5 and 5yrs.	Children’s daily screen-time at age 1 year (+), maternal country of birth (- for Australia vs other), maternal employment (-), longer sleep duration (-), attending child-care (-), having TV time rules (-), <2hrs screen time mother (-), TV on all the time (+), TV on during meals (+).
**Observational studies, longitudinal and cross-sectional analysis**				
Bernard et al. (2017), Singapore	861	24.4 ± 0.9mo, 47%	Q: screen time (hrs/day) at ages 2 and 3yrs.	Age (0), sex (0), Malay or Indian ethnicity vs Chinese (+), birth order (0), household income (0), maternal age (0), lower maternal education (+), paternal age (0), paternal education (0), paternal daily TV viewing at 24 and 36mo (0), parental marital status (0), maternal place of birth (0), private or public housing (0), and paternal BMI at 24 and 36mo (0)
			Q: TV time (hrs/day) at ages 2 and 3yrs.	Age (+), sex (0), Malay or Indian ethnicity vs Chinese (+), birth order (0), household income (0), maternal age (0), lower maternal education (+), maternal daily TV viewing > 3 h during pregnancy (+), paternal age(0), paternal education (0), paternal daily TV viewing at 24 and 36mo (0), parental marital status (0), maternal place of birth (0), private or public housing (0), and paternal BMI at 24 and 36mo (0)
			Q: hand-held device time (hrs/day) at ages 2 and 3yrs.	Age (+), sex (0), Malay or Indian ethnicity vs Chinese (+), birth order (+), household income (0), maternal age (0), lower maternal education (+), paternal age (0), paternal education (0), paternal daily TV viewing at 24 and 36mo (0), parental marital status (0), maternal place of birth (0), private or public housing (0), and paternal BMI at 24 and 36mo (0)
Detnakarintra et al. (2020), Thailand	280	2yrs, 53%	I: screen time on weekday (mins/day) at ages 2, 3 and 4yrs.	*Longitudinal analysis* Maternal education (- at ages 2 and 4yrs; 0 at age 3yrs), mother‐child interactions at 18mo (- at ages 2, 3 and 4yrs), nurturing parenting style at 3yrs (- at age 4yrs)
				*Cross-sectional analysis* Strict parenting style at age 3 (+)
Howe et al. (2017), New Zealand	487	24mo, 47%	Q: TV time (mins/day)	*Longitudinal analysis* Sex (0), ethnicity (0), estimated paternal BMI (0), maternal age (0), partner age (0), parental ethnicity (0), level of household deprivation (0)
				*Cross-sectional analysis* BMI z-score (0), first-born child (0), PA (0), maternal BMI (0), partner BMI (0), maternal screen time (+), maternal PA (0), Parenting style: authoritarian (+for mother and partner), authoritative (0 for mother and partner), and permissive parenting (+for mother; 0 for partner). Infant temperament: sociability (0 for mother and partner), emotionality (0 for mother and partner), activity (0 for mother and partner), attention span-persistence (0 for mother and partner), reaction to food (0 for mother and partner), and soothability (0 for mother and partner). Family type: active or sporty (0 for mother; - for partner), media savvy (0 for mother; + for partner), bookish (0 for mother and partner), outdoor people (0 for mother; - for partner), musical (0 for mother and partner), religious/ spiritual (0 for mother and partner), and creative or arty (0 for mother and partner).
**Observational studies, cross-sectional analysis**				
Abbott et al. (2015), Australia	432	4.6 ± 0.7yrs, 45%	Q: TV time (mins/day)	Sex (0), paternal TV viewing (+) and maternal TV viewing (+)
Alvarez et al. (2021), Chile, Colombia and Spain^a^	S1 = 496; S2 = 340; S3 = 437	S1 = 5.0, 45%; S2 = 5.0, 46%; S3 = 4.8, 74%	Q: TV time or playing video games (hrs/day)	Country (+for Chile vs Colombia and Spain), socio-economic level (0), parental education level (0), parental marital status (0)
Barr et al. (2010), USA^b^	S1 = 57; S2 = 51; S3 = 83; S4 = 48; S5 = 69	S1 = 198.9 ± 13.8dys; S2 = 292 ± 60.2dys; S3 = 382.4 ± 15.5dys; S4 = 459.4 ± 56.4dys; S5 = 566.9 ± 9.8dys, 46%	Q: TV exposure (hrs/day)	Age (0), gender (0), socioeconomic index (0), number of siblings (0), time use restrictions (0), and content restrictions (child content and no violence)(0), no-TV policy (0), season of the year (+for winter vs summer and fall), week vs weekend days (0)
Berglind et al. (2017), Sweden	899	4yrs, n.r.	Q: screen time on weekdays (mins/day)	Sex (0)
			Q: screen time on weekend days (mins/day)	Sex (0)
			Q: TV time on weekdays (mins/day)	Sex (0)
			Q: TV time on weekend days (mins/day)	Sex (0)
			Q: playing video games on weekdays (mins/day)	Sex (+for boys)
			Q: playing video games on weekend days (mins/day)	Sex (+for boys)
Birken et al. (2011), Canada	157	3yrs, 50%	Q: screen time on weekdays (mins/day)	Parental employment (+for maternal employment status), number of TVs in home (0), TV in bedroom child (0), TV in dining area (0), most common viewing-period (0), meals eaten with TV on (+for lunch and dinner; 0 for breakfast and snack), parental weekday screen time (0), household rules (0), parent watches with child (0).
			Q: screen time on weekend days (mins/day)	Parental employment (0), number of TVs in home (0), TV in bedroom child (0), TV in dining area (0), most common viewing-period (0), meals eaten with TV on (+for lunch, 0 for breakfast, dinner and snack), parental weekday screen time (+), household rules (-), parent watches with child (0); lunch in front of screen (+), parental screen time (+), family rules (-).
Bleakley et al. (2013), USA	465	0-5yrs, n.r.	Q: TV time (hrs/day)	Parent variables: parental TV viewing (+), co-viewing with child (+), parental restriction of child TV viewing (0), parental well-being (0), parental depression (0); media access variables: television in child’s bedroom (0), number of televisions in the home (0), computer with Internet access in child’s bedroom (0); demographic variables: child gender (0), child age (0), parental race (0), parental education (+for college degree or higher vs high, school education or less), parental income (0), parental employment status (+), number of children in the home (0)
Brown et al. (2010), Australia	2560	4-5yrs, 48%	Q: TV time (hrs/day)	Maternal high income (-), working part-time (-), weight status (+)
Carson et al. (2012), Canada	746	41 ± 16mo, 47%	Q: TV time and playing video/computer games (hrs/day)	Age (+), gender (- for girls), family income (-), parental self-efficacy (-), parental attitude (+), parental descriptive norms (+), parental screen time (+), TV in bedroom (+yes vs no), video games in bedroom (0)
Carson et al. (2014), Canada	551	0-5yrs, 45%	Q: screen time (mins/day)	Age (+), sex (0), child care status (0), family structure (0), parental education (-), neighborhood SES (-), walkability (0), Road speed (0), streetscape (0), outdoor play/activity space (0), recreation facilities (0), distance to closest park (0), yard space at home (0).
Carson et al. (2017), Canada	149	19 ± 1.9mo, 48%	Q: screen time (mins/day)	Age (+), Sex (+for girls), race/ethnicity (+other > European-Canadian/Caucasian), main type of child care (- child care center < parental, 0 for day home/other vs parental), number of siblings (0); parental characteristics - age (0), sex (0), parental marital status (0), country of birth (0), highest level of education (0), household income (+for $50,001 to $100,000 > >$100,000; 0 for ≤ $50,000 vs >$100,000).
			Q: TV time (mins/day)	Age (+), sex (0), race/ethnicity (+other > European-Canadian/Caucasian), main type of child care (- for child care center < parental, 0 for day home/other vs parental), number of siblings (0); parental characteristics - age (0), sex (0), parental marital status (0), country of birth (0), highest level of education (0), household income (+for $50,001 to $100,000 > >$100,000; 0 for ≤ $50,000 vs >$100,000).
			Q: playing video/computer games (mins/day)	Age (0), sex (0), race/ethnicity (+for other > European-Canadian/Caucasian), main type of child care (- for child care center/day home/other < parental), number of siblings (0); parental characteristics: age (0), sex (0), parental marital status (0), country of birth (0), highest level of education (0), household income (0).
Carson et al. (2020), Canada	1116	4yrs, 49%	Q: screen time (hrs/day)	Parental screen time (+)
Contreras et al. (2020), USA	572	4.1 ± 0.5yrs, 51%	Q: TV time and computer use (hrs/day)	Rural area (-)
Cowderoy et al. (2020), Australia	158	6.7 ± 0.8mo, 45%	Q: screen time (hrs/day)	Maternal depression: no depression (0), past depression (0), current depression(0)
De Craemer et al (2015), Belgium, Bulgaria, Germany, Greece, Poland and Spain	8117	4.8 ± 0.5yrs, 47%	Q: TV time on weekdays (mins/day)	Country differences (+for Germany < Spain < Belgium < Poland < Bulgaria < Greece)
			Q: TV time on weekend days (mins/day)	Country differences (+for Germany vs Poland vs Belgium vs Spain vs Bulgaria vs Greece)
			Q: computer use on weekdays (mins/day)	Country differences (+for Germany vs Spain vs Belgium vs Poland vs Greece vs Bulgaria)
			Q: computer use on weekend days (mins/day)	Country differences (+for Germany vs Belgium vs Greece vs Spain vs Poland vs Bulgaria)
De Decker et al. (2015), Australia and Belgium^c^	S1 = 947; S2 = 1527	S1 = 4.5 ± 0.7yrs, 46%; S2 = 4.4 ± 0.6yrs, 53%	Q: TV time (hrs/day)	Parental TV viewing time (+), parental concerns about screen viewing (+only in Australian sample; 0 for Belgium sample), parental rules about TV viewing (-)
Downing et al. (2017), Australia	937	4.5 ± 0.7yrs, 46%	Q: screen time and sedentary electronic game use on weekdays and weekend days (mins/day)	BMI (0), disability/poor health (0), parents live together (0), sleep duration (-), siblings (0), active transport (- for girls; 0 for boys), non-organized activities (0), organized activities (- for girls; 0 for boys), attending playgroup (0), outdoor play time (0), maternal age (0), paternal age (0), mother born in Australia (-), father born in Australia (0 for boys; - for girls), maternal BMI (0), paternal BMI (0), maternal education (0), paternal education (0), mother in paid employment (0), father in paid employment (0), low income status (0 for boys; + for girls), maternal disability/poor health (0), paternal disability/poor health (0), maternal time in PA (0), paternal time in PA (0 for boys; - for girls), maternal TV viewing (+), paternal TV viewing (+), maternal role modelling of PA (0 for boys; - for girls), paternal role modelling of PA (0 for boys; - for girls), dog ownership (0), pieces of toys/ equipment to be physically active with at home (0 for boys; - for girls), lives on medium/large block (0), number of features at home (-), lives on a cul-de-sac (+for boys; 0 for girls), pieces of electronic equipment at home (0), number of TVs at home (+), TV in child’s bedroom (+), computer/e-games in child’s bedroom (0), neighborhood playground suitability (-), neighborhood constraints to active transport (0 for boys; - for girls), total frequency of visiting active places per week (0) Child personality, preferences and constraints: active co-participation preferences (0), active for longer with someone else (- for boys; 0 for girls), competitive with other children when being active (0), prosocial PA behavior (0), preferences for SB (+), constraints to PA (+for boys; 0 for girls); social interaction and support: child is active at social gatherings (0), maternal PA emotional support for child (+for boys; 0 for girls), paternal PA emotional support for child (+for boys; 0 for girls); Parental influence: concerns about child’s PA/SB (+), constraints to child’s PA (+), likes to participate in outdoor play (0), prefers to be social with other parents (0), gets bored watching child playing in outdoor spaces (- for boys; 0 for girls), likes child to do activities of older children (0), likes child to do activities they did as a child (0), gets bored going to the same place (0), believes it’s important to be active as a family (- for boys; 0 for girls), self-efficacy to support PA (-), self-efficacy to limit screen time (-), health knowledge/beliefs of child’s PA (0 for boys; - for girls) Rules and boundaries: rules to limit screen time (-), rules about games inside (0), rules about PA for stranger danger, traffic, injury (0), allows child to play freely in backyard/street (0 for boys; - for girls), switches off screen entertainment (0)
Flores et al. (2005), USA^d^	S1 = 718; S2 = 477; S3 = 817	S1 = 19.5mo, 47%; S2 = 19.5mo, 52%; S3 = 18.7mo, 49%	I: TV time (hrs/day)	Racial/Ethnic disparities (0)
French et al. (2017), USA	534	2-4yrs, 51%*	Q: TV time on weekdays (hrs/day)	Park visits (0)
			Q: TV time on weekend days (hrs/day)	Park visits (-)
Hinkley et al. (2017), Australia	575	3.8yrs, 46%	Q: screen time (hrs/day)	Maternal perception of impact of screen time on health/development: physical wellbeing (0), cognitive wellbeing (-), social wellbeing (0)
Khan et al. (2017), USA	S1 = 8900, S2 = 506	S1 = 4.4 ± 0.4yrs, 51%; S2 = 4.3 ± 0.5yrs, 54%	Q: TV time on typical weekday (hrs/day)	Frequency of parent–child shared reading (- for both samples)
Kim et al. (2021), Korea	352	2-5yrs, 48%	Q: smartphone usage (n.r.)	Maternal smartphone addiction (0), maternal employment status (0), maternal parenting stress index (0), maternal beck depression inventory (0), maternal beck anxiety inventory (0)
Lee et al. (2018), Canada	193	1.6 ± 0.2yrs, 51%	Q: screen time (mins/day)	Electronic device in the bedroom (+), neighborhood safety (0), parental modelling (+), limit on screen time (-), positive outcome expectations (0), negative outcome expectations (0), barrier self-efficacy (0)
Lee et al. (2020), Canada en Korea^e^	S1 = 121, S2 = 101	S1 = 3.4 ± 0.7yrs, 50%; S2 = 3.4 ± 1.2yrs, 55%	Q: screen time (mins/day)	Positive outcome expectations (0), negative outcome expectations (+), barrier self-efficacy (-), parental screen time (+), parental limit on child’s screen time (0), screen-based electronic equipment at home (0), screen-based electronic equipment in child’s bedroom (+), neighborhood safety (-)
Leppanen et al. (2020), Finland	779	4.7 ± 0.9yrs, 48%	7 day Diary: screen time (mins/day)	Child’s long-term stress (0), child’s temperament: effortful control (-), negative affectivity (0), surgency (0)
Maatta et al. (2017), Finland	771	4.7 ± 0.9yrs, 48%	Diary: screen time at home (min/day)	Maternal education (+for low or medium vs high), paternal education (+for low vs high), household income (+for low vs high)
Maatta et al. (2017), Finland	768	4.7 ± 0.9yrs, 49%	Diary: screen time at home (min/day)	Access to screens at home (+), descriptive norm for screen time (+), parental satisfaction of children’s screen time (-), rules for limiting screen time (+), parental screen time in front of children (+), parental importance for limiting screen time (-), parental attitude toward societal pressures for screen time (+), parental self-efficacy for limiting screen time (+).
Morowatisharifa et al. (2015), Iran	188	4.1 ± 0.7yrs, 51%	Q: TV time (hrs/day)	Sex (0), age group (+), BMI group (-), paternal education (0), maternal education (0), father with child (0), mother with child (0), father watching TV (0), mother watching TV (+), Size of the house (0), Having a yard (-)
Nikken et al. (2015), The Netherlands	896	3.4 ± 2.3yrs, 51%	Q: TV time (mins/day)	Age (+), gender (0), more media skills (+), parental attitudes about media: positive effects (0), negative effects (0), used for pacifying (0), too complicated (0); increased parental media use (+), parental gender (0), higher parental educational level (-), higher family income (0), family size (0)
			Q: time spent on game devices (mins/day)	Age (+), gender (- for boys), more media skills (+), parental attitudes about media: positive effects (0), negative effects (0), used for pacifying (0), too complicated (0); increased parental media use (+), parental gender (0), higher parental educational level (0), higher family income (0), family size (0)
			Q: time spent on computers (mins/day)	Age (+), gender (0), more media skills (+), parental attitudes about media: positive effects (0), negative effects (0), used for pacifying (+), too complicated (0); increased parental media use (+), parental gender (0), higher parental educational level (-), higher family income (-), family size (0)
			Q: time spent on touchscreens (mins/day)	Age (0), gender (0), more media skills (+), parental attitudes about media: positive effects (0), negative effects (0), used for pacifying (+), too complicated (0); increased parental media use (+), parental gender (0), higher parental educational level (0), higher family income (-), family size (0)
Njoroge et al. (2013), USA^f^	S1 = 409; S2 = 41; S3 = 49; S4 = 97	S1 = 4.3 ± 0.6yrs, 46%; S2 = 4.3 ± 0.7yrs, 39%; S3 = 4.3 ± 0.7yrs, 47%; S4 = 4.2 ± 0.7yrs, 41%	Diary: TV time (mins/wk)	Race/ethnicity (0), Education-income matrix (0), sex (0), TV in bedroom child (+), only child (+), average time in primary child care(0), “My child’s other caregiver supports me in reducing TV for my child” and expects positive effects from educational TV (+for agree to both statements vs disagree/neutral to both statements; 0 for agree to at least one statement), confidence in ability to limit exposure to TV (-)
Rodrigues et al. (2020), Portugal	2397	4.5 ± 0.7yrs, 49%	Q: screen time (mins/day)	Socio-economic position (-)
			Q: screen time on weekdays (mins/day)	Socio-economic position (0)
			Q: screen time on weekend days (mins/day)	Socio-economic position (-)
			Q: TV time on weekdays (mins/day)	Socio-economic position (0)
			Q: TV time on weekend days (mins/day)	Socio-economic position (0)
			Q: computer time on weekdays (mins/day)	Socio-economic position (0)
			Q: computer time on weekend days (mins/day)	Socio-economic position (0)
			Q: electronic games time on weekdays (mins/day)	Socio-economic position (0)
			Q: electronic games time on weekend days (mins/day)	Socio-economic position (0)
			Q: smartphone time on weekdays (mins/day)	Socio-economic position (0)
			Q: smartphone time on weekend days (mins/day)	Socio-economic position (0)
			Q: tablet time on weekdays (mins/day)	Socio-economic position (0)
			Q: tablet time on weekend days (mins/day)	Socio-economic position (0)
Rodriques et al. (2021), Portugal	2397	3-5yrs, 48%	Q: screen time on weekdays (mins/day)	Television in bedroom (+for boys and girls), computer in bedroom (0 for boys and girls), laptop in bedroom (+for boys; 0 for girls), tablet in bedroom (+for boys and girls)
			Q: screen time on weekend days (mins/day)	Television in bedroom (+for boys and girls), computer in bedroom (0 for boys and girls), laptop in bedroom (+for boys; 0 for girls), tablet in bedroom (+for boys and girls)
Sanders et al. (2016), USA	210	4.8 ± 1.3yrs, 47%	Q: screen time (hrs/wk)	Parental negative attitudes about technology (0), parent’s perceived efficacy (-), technology-related parenting strategies (-)
Sijtsma et al. (2015), The Netherlands	759	3.9 ± 0.1yrs, 47%	Q: screen time (mins/day)	TV in bedroom (+), number of TVs (+)
			Q: screen time on weekdays (mins/day)	TV in bedroom (+), number of TVs (+)
			Q: screen time on weekend days (mins/day)	TV in bedroom (+), number of TVs (+)
Tang et al. (2018), Canada	62	3.7 ± 1.4yrs, 44%	Q: recreational screen time on weekdays (hrs/day)	Screen time modeling (+for mother; 0 for father), mealtime screen use (+for mother and father), screens to control behavior (+for mother; 0 for father), monitoring screen time (- for mother and father), limiting screen time (- for mother and father), weekday screen time (0 for mother and father).
			Q: recreational screen time on weekend days (hrs/day)	Screen time modeling (0 for mother and father), mealtime screen use (0 for mother and father), screens to control behavior (+for mother and father), monitoring screen time (- for mother and father), limiting screen time (- for mother and father), weekday screen time (0 for mother and father).
Thompson et al. (2007), USA	1793	S1 = 4-11mo; S2 = 12-23mo; S3 = 24-36mo, 49%	I: screen time (hrs/day)	Age (0), maternal race/ethnicity (0), maternal education (0), income (0), maternal mental distress (+), number of children in the house (0)
Thompson et al. (2010), USA	1332	S1 = 4-11mo; S2 = 12-23mo; S3 = 24-36mo, n.r.	I: TV time (hrs/day)	Age (+), race/ethnicity (0), maternal language preference S1 (0), maternal language preference S2 (+for English speaking Hispanic mothers vs Spanish-speaking Hispanic mothers), maternal language preference S3 (+for English speaking Hispanic mothers vs Spanish-speaking Hispanic mothers)
Thompson et al. (2018), USA	312	3.9 ± 0.8yrs, 46%	I: TV time (mins/day)	Parenting practices: time restriction (-), TV in child’s bedroom (+), allowing viewing with meals (0), allowing viewing with snacks (+)
Tombeau et al. (2020), Canada	1085	11.6 ± 2.3mo, 48%	Q: screen time (min/wk)	Parenting stress (+), child age (0), child negative affectivity (0), family living arrangements (2 parents in the same household or single parent/other type of household)(0), and family income (+for less than $60 000 > equal or more than $60 000)
Vaala et al. (2014), USA	696	3-24mo, 51%	Q: TV time (hrs/wk)	Structural life circumstances: child age (+), having another child aged 3–24mo (0), toy index (+), number of non-traditional sources for the child's video-viewing (+), having a television in the child's bedroom (0), maternal unemployment (+), maternal TV/video viewing (+), childcare TV/videos use (+), use of childcare (-); attitude (+), injunctive norms (0), descriptive norms (+), perceived behavioral control (-)
Waller et al. (2021), USA	1700	4.4 ± 0.6yrs, 49%	Q: screen time (hrs/wk)	age (+), sex (+boys), black race (+black > non-black), parental income (-), parental education (+)
Wang et al. (2020), China	1772	3-6yrs, 47%	Q: screen time (mins/day)	Urban vs rural (-); Neighborhood environments: many places to buy things within easy walking distance of home (0), within a 10- to 15-min walk to transit stop from home (0), sidewalks (0), facilities to bicycle (0), Free or low-cost recreation facilities (0), high crime rate (0), much traffic (+for urban, 0 for rural), many physically active people (0), many interesting things around (0), motor vehicles in household? (0); Physical home environments: TV or computer in child’s room (0), rules around electronic devices (0), amount of media devices at home? (+for urban; 0 for rural); social-culture home environments: outdoor play limited due to fear for injuries (+), academic abilities more important than physical development (+), other people should be present for child’s outdoor play (+)
Wiseman et al. (2019), Australia	138	49.9 ± 5.4mo, 48%	Q: screen time (hrs/wk)	Child PA preference (0), child PA knowledge (0); Controlling factors: rules indoor play (0), rules outdoor play (+), PA as reward/to control behavior (0), limiting/monitoring screen-time (-), limiting outdoor play due to weather (0), use of screen-time to reward/control child behavior (0); Promoting factors: explicit modelling and enjoyment of PA (+), verbal encouragement (0), instrumental support for sport (-), instrumental support for active play (0), importance and value PA (-), support/reinforcement from other adults (-), exposure to TV (+), explicit modelling and enjoyment of screen time (+)
			Q: screen time on weekdays (hrs/5 wk days)	Child PA preference (0), child PA knowledge (0); Controlling factors: rules indoor play (0), rules outdoor play (+), PA as reward/to control behavior (0), limiting/monitoring screen-time (-), limiting outdoor play due to weather (0), use of screen-time to reward/control child behavior (0); Promoting factors: explicit modelling and enjoyment of PA (0), verbal encouragement (0), instrumental support for sport (0), instrumental support for active play (0), importance and value PA (0), support/reinforcement from other adults (-), exposure to TV (+), explicit modelling and enjoyment of screen time (+)
			Q: screen time on weekend days (hrs/weekend)	Child PA preference (0), child PA knowledge (0); Controlling factors: rules indoor play (0), rules outdoor play (+), PA as reward/to control behavior (0), limiting/monitoring screen-time (-), limiting outdoor play due to weather (0), use of screen-time to reward/control child behavior (0); Promoting factors: explicit modelling and enjoyment of PA (+), verbal encouragement (0), instrumental support for sport (-), instrumental support for active play (0), importance and value PA (-), support/reinforcement from other adults (-), exposure to TV (+), explicit modelling and enjoyment of screen time (+)

Abbreviations: BMI = Body Mass Index, CON = control, g = gram, hrs = hours, INT = intervention, I = interview, kg = kilogram, min = minutes, mo = months, n.r. = not reporte, PA = physical activity, Q = questionnaire, S = sample, SB = sedentary behavior, TV = television, wk(s) = week(s), yrs = years.

a Samples defined as S1 = Colombia, S2 = Chile, S3 = Spain; ^b^ Samples defined as S1 = 6mo, S2 = 9mo, S3 = 12mo, S4 = 15mo, S5 = 18mo; ^c^ Samples defined as S1 = Australia, S2 = Belgium; ^d^ Samples defined as S1 = white children, S2 = black children, S3 = Hispanic children; ^e^ Samples defined as S1 = Canada, S2 = Korea; ^f^ Samples defined as S1 = Non-Hispanic White, S2 = African American, S3 = Asian American/PI/Hawaiian, S4 = Multiracial/other; * = Weighted average across groups;

In total, longitudinal analyses were included from 11 studies (Barber, 2017, Bernard, 2017, Detnakarintra, 2020, Hish, 2021, Hnatiuk, 2015, Howe, 2017, Krogh, et al., xxxx, Matarma, 2016, Thompson, 2015, Xu, 2016) and cross-sectional analyses from 45 studies (Bernard, 2017, Detnakarintra, 2020, Howe, 2017, Abbott, 2016, Álvarez, et al., xxxx, Barr, 2010, Berglind and Tynelius, 2017, Birken, 2011, Bleakley et al., 2013, Brown, 2010, Carson and Janssen, 2012, Carson and Kuzik, 2017, Carson et al., 2020, Carson et al., 2014, Contreras, et al., xxxx, Cowderoy, 2020, De Craemer, 2015, De Decker, 2015, Downing, 2017, Flores et al., 2005, French, 2017, Hinkley, 2017, Khan, 2017, Kim, et al., xxxx, Lee, 2018, Lee, 2021, Leppänen, 2020, Määttä, 2017, Määttä, 2017, Morowatisharifabad et al., 2015, Nikken and Schols, 2015, Njoroge, 2013, Rodrigues, et al., xxxx, Rodrigues, 2020, Sanders, 2016, Sijtsma, 2015, Tang, 2018, Thompson and Christakis, 2007, Thompson, 2018, Thompson, 2010, Tombeau Cost, 2020, Vaala and Hornik, 2014, Waller, 2021, Wang, 2020, Wiseman et al., 2019). Sample sizes ranged from 62 to 10,700. Most studies included between 501 and 1000 participants (n = 18 studies) (Bernard, 2017, Matarma, 2016, Thompson, 2015, Álvarez, et al., xxxx, Berglind and Tynelius, 2017, Carson and Janssen, 2012, Carson et al., 2014, Contreras, et al., xxxx, Downing, 2017, French, 2017, Hinkley, 2017, Leppänen, 2020, Määttä, 2017, Määttä, 2017, Nikken and Schols, 2015, Njoroge, 2013, Sijtsma, 2015, Vaala and Hornik, 2014), and 17 studies included>1000 participants (Barber, 2017, Krogh, et al., xxxx, Álvarez, et al., xxxx, Brown, 2010, Carson et al., 2020, De Craemer, 2015, De Decker, 2015, Flores et al., 2005, Khan, 2017, Rodrigues, et al., xxxx, Rodrigues, 2020, Thompson and Christakis, 2007, Thompson, 2010, Tombeau Cost, 2020, Waller, 2021, Wang, 2020, Morrissey, 2014). Most studies were conducted among children with a mean age between 4 and 5 years (n = 19 studies) (Abbott, 2016, Álvarez, et al., xxxx, Berglind and Tynelius, 2017, Brown, 2010, Carson et al., 2020, Contreras, et al., xxxx, De Craemer, 2015, De Decker, 2015, Downing, 2017, Khan, 2017, Leppänen, 2020, Määttä, 2017, Määttä, 2017, Morowatisharifabad et al., 2015, Njoroge, 2013, Rodrigues, 2020, Sanders, 2016, Waller, 2021, Wiseman et al., 2019), while four studies were conducted in children aged 2-to-3-years (Bernard, 2017, Detnakarintra, 2020, Hish, 2021, Howe, 2017). Six studies were conducted in children under age 1 (Barber, 2017, Hnatiuk, 2015, Krogh, et al., xxxx, Cowderoy, 2020, Tombeau Cost, 2020, Morrissey, 2014), five in children aged 1–2 years (Matarma, 2016, Xu, 2016, Carson and Kuzik, 2017, Flores et al., 2005, Lee, 2018), nine studies in children aged 3–4 years (Thompson, 2015, Birken, 2011, Carson and Janssen, 2012, Hinkley, 2017, Lee, 2021, Nikken and Schols, 2015, Sijtsma, 2015, Tang, 2018, Thompson, 2018) and ten studies across a wider age range (Barr, 2010, Bleakley et al., 2013, Carson et al., 2014, French, 2017, Kim, et al., xxxx, Thompson and Christakis, 2007, Thompson, 2010, Vaala and Hornik, 2014, Wang, 2020, Rodrigues, 2021). Most studies were conducted in North America (n = 23 studies) (Hish, 2021, Thompson, 2015, Barr, 2010, Birken, 2011, Bleakley et al., 2013, Carson and Janssen, 2012, Carson and Kuzik, 2017, Carson et al., 2020, Carson et al., 2014, Contreras, et al., xxxx, Khan, 2017, Lee, 2018, Lee, 2021, Njoroge, 2013, Sanders, 2016, Tang, 2018, Thompson and Christakis, 2007, Thompson, 2018, Thompson, 2010, Tombeau Cost, 2020, Vaala and Hornik, 2014, Waller, 2021, Morrissey, 2014), followed by Europe (n = 16 studies) (Barber, 2017, Krogh, et al., xxxx, Matarma, 2016, Álvarez, et al., xxxx, Berglind and Tynelius, 2017, De Craemer, 2015, De Decker, 2015, Flores et al., 2005, French, 2017, Leppänen, 2020, Määttä, 2017, Määttä, 2017, Nikken and Schols, 2015, Rodrigues, et al., xxxx, Rodrigues, 2020, Sijtsma, 2015).. Forty-two studies reported on total screen time (e.g., hours or minutes per day or week) and twelve studies used separate analyses for different outcomes of screen time (e.g., week and weekend days).

In total, 91 different correlates of screen time were assessed. Eight correlates were classified as biological correlates, six as behavioral attributes and skills, one as cognitive, emotional or psychological correlate, seven as physical environmental correlates, seven as economic correlates, one as political correlate and 61 as sociocultural correlates. Forty-four correlates were only examined once (in only one study) and as such we could only conclude insufficient evidence. Some of these studies examined correlates that were only examined in that study (Bernard, 2017, Hish, 2021, Howe, 2017, Matarma, 2016, Xu, 2016, Barr, 2010, Birken, 2011, Downing, 2017, Khan, 2017, Kim, et al., xxxx, Leppänen, 2020, Määttä, 2017, Nikken and Schols, 2015, Tang, 2018, Thompson, 2010, Tombeau Cost, 2020, Vaala and Hornik, 2014, Wiseman et al., 2019). These correlates are not further discussed. Table 2 presents the summary of results regarding correlates of screen time examined in at least 2 studies.

**Table 2:** Summary of results regarding correlates of screen time in young children (<4 years).

**Correlate**	**Included studies**^a^**^,b^**	**Summary coding** **(n/N, (%))**^a^**^,b^**	**Evidence synthesis**
***Biological correlates***			
Sex	+ + **0** 0 0 0 0 0 0 0 0 0 0 0 -	**0 (12/15, 80%)**	Based on consistent findings among studies with low-to-high methodological quality, there is no significant evidence for an association between sex and screen time.
Age	**+** + + + + + + + 0 0 0 0 0	?	Based on inconsistent findings among studies with low-to-high methodological quality, there is insufficient evidence for an association between age and screen time.
Ethnicity/race child or parent/country of birth parent	+ + + + + **0** 0 0 0 0 - - -	?	Based on inconsistent findings among studies with low-to-high methodological quality, there is insufficient evidence for an association between ethnicity/race of the child or parent and screen time.
Siblings/nr of children in the house	0 0 0 0 0 0 0 -	**0 (7/8, 88%)**	Based on consistent findings among studies with low-to-moderate methodological quality, there is no significant evidence for an association between having siblings/the number of children in the house and screen time.
First-born	+ 0 0 0	**0 (3/4, 75%)**	Based on consistent findings among studies with low-to-moderate methodological quality, there is no significant evidence for an association between being first-born and screen time.
BMI	0 0 0 -	**0 (3/4, 75%)**	Based on consistent findings among studies with low-to-moderate methodological quality, there is no significant evidence for an association between BMI and screen time.
***Behavioral attributes and skills-related correlates***			
Sleep duration	- -	**- (2/2, 100%)**	Based on consistent findings among studies with low-to-moderate methodological quality, there is moderate evidence for a negative association between sleep duration and screen time.
Physical activity	0 0	**0 (2/2, 100%)**	Based on consistent finding among studies with low-to-moderate methodological quality, there is no significant evidence for an association between physical activity and screen time.
***Cognitive, emotional or psychological correlates***			
Temperament/personality	0 0 0	**0 (3/3, 100%)**	Based on consistent findings among studies with low-moderate methodological quality, there is in no significant evidence for an association between temperament/personality and screen time.
***Physical environmental correlates***			
Electronic devices/computer/ TV in (bed)room where child sleeps	+ + + + + + + + + 0 0 0	**+ (9/12, 75%)**	Based on consistent findings among studies with low-to-moderate methodological quality, there is moderate evidence for a positive association between having electronic devices in the (bed) room where the child sleeps and screen time.
Electronic devices/screen-based/TVs at home	+ + + + 0 0 0	?	Based on inconsistent findings among studies with low-to-moderate methodological quality, there is insufficient evidence for an association between having electronic devices at home and screen time.
House features (size, presence yard, fences, covered outdoor area)	- -	**- (2/2), 100%)**	Based on consistent findings among studies with low-to-moderate methodological quality, there is moderate evidence for a negative association between house features and screen time.
Neighbourhood-related factors	0 0 0 0 -	**0 (4/5, 80%)**	Based on consistent findings among studies with low-to-moderate quality methodological quality, there is no significant evidence for an association between neighborhood-related factors and screen time.
Toys availability	+ -	?	Based on inconsistent findings among studies with low-to-moderate methodological quality, there is insufficient evidence for an association between having toys and screen time.
***Economic correlates***			
Parental education	0 0 0 0 0 0 - - - - - - - - - -	?	Based on inconsistent findings among studies with low-to-moderate methodological quality, there is insufficient evidence for an association between parental education and screen time.
Family income	0 0 0 0 0 0 0 0 - - - - - - - -	?	Based on inconsistent findings among studies with low-to-moderate methodological quality, there is insufficient evidence for an association between family income and screen time.
Socio-economic variables	0 0 0 0	**0 (4/4, 100%)**	Based on consistent findings among studies with low-to-moderate methodological quality, there is no significant evidence for an association between socio-economic variables and screen time.
Parental employment	+ + + 0 0 - - -	?	Based on inconsistent findings among studies with low-to-moderate methodological quality, there is insufficient evidence for an association between parental employment and screen time.
Rural vs urban living area	- -	**- (2/2, 100%)**	Based on consistent findings among studies with low-to-moderate methodological quality, there is moderate evidence for a negative association between living area and screen time.
Marital status/parents live together	0 0 0 0 0 0	**0 (6/6, 100%)**	Based on consistent findings among studies with low-to-moderate methodological quality, there is no significant evidence for an association between marital status/parents living together and screen time.
***Sociocultural correlates***			
Parental screen time/media use/modelling	+ + + + + + + + + + + + + + + + + 0 0 0	**+ (17/20, 85%)**	Based on consistent findings among studies with low-to-moderate methodological quality, there is moderate evidence for a positive association between parental screen time and children’s screen time.
Rules around screen time	+ 0 0 0 0 - - - - - - - - -	?	Based on inconsistent findings among studies with low-to-moderate methodological quality, there is insufficient evidence for an association between having rules around screen time and screen time.
TV on at home	+ + +	**+ (3/3, 100%)**	Based on consistent findings among studies with low-to-moderate methodological quality, there is moderate evidence for a positive association between having a TV at home and screen time.
Monitoring screen time	- -	**- (2/2, 100%)**	Based on consistent findings among studies with low-to-moderate methodological quality, there is moderate evidence for a negative association between monitoring screen time and children’s screen time.
Away from home care	0 0 - - - - -	**- (6/8, 75%)**	Based on consistent findings among studies with low-to-moderate methodological quality, there is moderate evidence for a negative association between being away from home care and screen time.
Placing high importance and value on physical activity	- -	**- (2/2, 100%)**	Based on consistent findings among studies with low-to-moderate methodological quality, there is moderate evidence for a negative association between placing high importance and value on physical activity and screen time.
Descriptive norms	+ + +	**+ (3/3, 100%)**	Based on consistent findings among studies with low-to-moderate methodological quality, there is moderate evidence for a positive association between descriptive norms and screen time.
TV on during meals/snacks	+ + 0 0	?	Based on inconsistent findings among studies with low-to-moderate methodological quality, there is insufficient evidence for an association between having a TV on during meals and screen time.
Parental physical activity	0 0	**0 (2/2, 100%)**	Based on consistent findings among studies with low-to-moderate methodological quality, there is no significant evidence for an association between parental physical activity and screen time.
Park visits/active places	0 -	?	Based on inconsistent findings among studies with low-to-moderate methodological quality, there is insufficient evidence for an association between park visits and screen time.
Parental weight status	+ 0 0 0 0	**0 (4/5, 80%)**	Based on consistent findings among studies with low-to-moderate methodological quality, there is no significant evidence for an association between parental weight status and screen time.
TV on to control behaviour	+ 0	?	Based on inconsistent findings among studies with low-to-moderate methodological quality, there is insufficient evidence for an association between having a TV on to control behavior and screen time.
Support/reinforcement for physical activity from other adults	+ -	?	Based on inconsistent findings among studies with low-to-moderate methodological quality, there is insufficient evidence for an association between support for physical activity from other adults and screen time.
Parental sex	0 0	**0 (2/2, 100%)**	Based on consistent findings among studies with low-to-moderate methodological quality, there is no significant evidence for an association between parental sex and screen time.
Parent-child co-watching	+ -	?	Based on inconsistent findings among studies with low-to-moderate methodological quality, there is insufficient evidence for an association between parent–child co-watching and screen time.
Parent-child interactions/time with child	0 -	?	Based on inconsistent findings among studies with low methodological quality, there is insufficient evidence for an association between parent–child interactions and screen time.
Parental health and wellbeing	0 0	**0 (2/2, 100%)**	Based on consistent findings among studies with low-to-moderate methodological quality, there is no significant evidence for an association between parental health and well-being and screen time.
Positive outcome expectations	0 0	**0 (2/2, 100%)**	Based on consistent findings among studies with low-to-moderate methodological quality, there is no significant evidence for an association between positive outcome expectations and screen time.
Parental depression	+ 0 0 0	**0 (3/4, 75%)**	Based on consistent findings among studies with low-to-moderate methodological quality, there is no significant evidence for an association between parental depression and screen time.
Parental mental health: stress	+ + 0 0	?	Based on inconsistent findings among studies with low-to-moderate methodological quality, there is insufficient evidence for an association between parental mental health and screen time.
Parental age	+ 0 0 0 0 0	**0 (5/6,83%)**	Based on consistent findings among studies with low-to-moderate methodological quality, there is no significant evidence for an association between parental age and screen time.
Parenting styles/strategies	+ -	?	Based on inconsistent findings among studies with low methodological quality, there is insufficient evidence for an association between parenting styles and screen time.
Parental attitudes	+ + + + + + 0 0 0	?	Based on inconsistent findings among studies with low-to-moderate methodological quality, there is insufficient evidence for an association between parental attitudes and screen time.
Negative outcome expectations	+ 0	?	Based on inconsistent findings among studies with low-to-moderate methodological quality, there is insufficient evidence for an association between negative outcome expectations and screen time.
Confidence to limit TV time	0 -	?	Based on inconsistent findings among studies with low-to-moderate methodological quality, there is insufficient evidence for an association between the confidence to limit TV time and screen time.
Parental self-efficacy	0 - - - - - -	**- (6/7,86%)**	Based on consistent findings among studies with low-to-moderate methodological quality, there is moderate evidence for a negative association between parental self-efficacy and screen time.
Country	+ +	**+ (2/2, 100%)**	Based on consistent findings among studies with low-to-moderate methodological quality, there is moderate evidence for a positive association between countries and screen time.

a bold indicates results from high quality study; ^b^ summary score: + = consistent positive association, - = consistent negative association, 0 = consistent no association,? = inconsistent findings; score is based on all studies unless 2 or more high quality studies were available. Abbreviations: BMI = body mass index, PA = physical activity, TV = television.

### 3.2 Overview of methodological quality

**Fig. 2:**
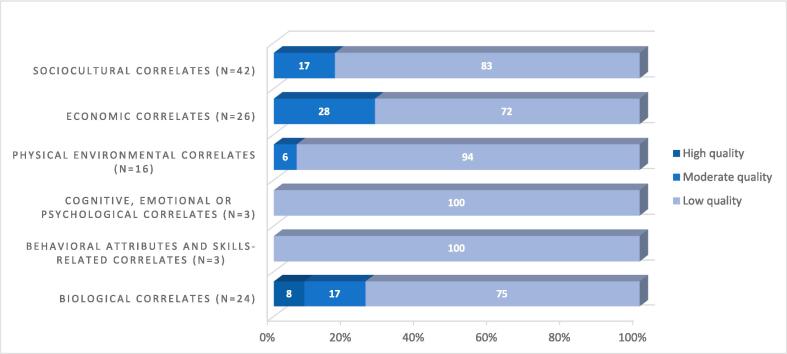
Methodological quality.

Two out of 53 studies were rated as high methodological quality (Berglind and Tynelius, 2017, Thompson, 2010), one study as high-to-moderate quality (Bernard, 2017), nine studies as moderate quality (Krogh, et al., xxxx, Álvarez, et al., xxxx, Birken, 2011, Contreras, et al., xxxx, De Decker, 2015, Flores et al., 2005, Määttä, 2017, Thompson and Christakis, 2007, Tombeau Cost, 2020) and forty-three studies as moderate-to-low or low methodological quality (Barber, 2017, Detnakarintra, 2020, Hish, 2021, Hnatiuk, 2015, Howe, 2017, Matarma, 2016, Thompson, 2015, Xu, 2016, Abbott, 2016, Barr, 2010, Bleakley et al., 2013, Brown, 2010, Carson and Janssen, 2012, Carson and Kuzik, 2017, Carson et al., 2020, Carson et al., 2014, Cowderoy, 2020, De Craemer, 2015, Downing, 2017, French, 2017, Hinkley, 2017, Khan, 2017, Kim, et al., xxxx, Lee, 2018, Lee, 2021, Leppänen, 2020, Määttä, 2017, Morowatisharifabad et al., 2015, Nikken and Schols, 2015, Njoroge, 2013, Rodrigues, et al., xxxx, Rodrigues, 2020, Sanders, 2016, Sijtsma, 2015, Tang, 2018, Thompson, 2018, Vaala and Hornik, 2014, Waller, 2021, Wang, 2020, Wiseman et al., 2019, Morrissey, 2014). Fig. 2 and supplementary file 4 shows the methodological quality of include studies.

### 3.3 Personal correlates

#### 3.3.1 Biological correlates

Twenty-four studies examined biological correlates, of which sex was most frequently examined (n = 15 studies), followed by age and race/ethnicity (both n = 13 studies). The majority of studies found no significant association between sex and screen time (n = 12 studies) (Bernard, 2017, Howe, 2017, Matarma, 2016, Abbott, 2016, Barr, 2010, Berglind and Tynelius, 2017, Bleakley et al., 2013, Carson and Kuzik, 2017, Carson et al., 2014, Morowatisharifabad et al., 2015, Nikken and Schols, 2015, Njoroge, 2013). Results were inconsistent for age and race/ethnicity. Eight out of 13 studies examining age found that a higher age was associated with more screen time, while five studies found no significant association (Bernard, 2017, Carson and Janssen, 2012, Carson and Kuzik, 2017, Carson et al., 2014, Morowatisharifabad et al., 2015, Thompson, 2010, Vaala and Hornik, 2014, Waller, 2021). Eight studies examining race/ethnicity of either the child or parent found a significant association with screen time (Barber, 2017, Bernard, 2017, Hish, 2021, Xu, 2016, Carson and Kuzik, 2017, Downing, 2017, Thompson, 2010, Waller, 2021) and five studies found no significant association (Howe, 2017, Bleakley et al., 2013, Flores et al., 2005, Njoroge, 2013, Thompson and Christakis, 2007). Seven out of eight studies found no significant association between having siblings/the number of children at home and screen time (Barr, 2010, Bleakley et al., 2013, Carson and Kuzik, 2017, Downing, 2017, Nikken and Schols, 2015, Thompson and Christakis, 2007, Vaala and Hornik, 2014) and three out of four studies found no significant associations between being a first-born child and screen time (Bernard, 2017, Howe, 2017, Matarma, 2016). One study found that a lower BMI was associated with less screen time (Matarma, 2016) whereas three studies found no significant association (Howe, 2017, Downing, 2017, Morowatisharifabad et al., 2015).

In summary, we found no evidence for an association of child sex, BMI, the number of siblings/children at home and being a first-born child with screen time. We found inconsistent evidence for an association of age and race/ethnicity with screen time.

#### 3.3.2 Behavioral attributes and skills correlates

Three studies examined behavioral attributes and skills-related correlates. Two studies examining sleep duration found a negative association with screen time (Xu, 2016, Downing, 2017). Two studies examining physical activity found no significant association with screen time (Howe, 2017, Downing, 2017). In summary, we found moderate evidence for a longer sleep duration being associated with less screen time and we found no evidence for an association of physical activity with screen time.

#### 3.3.3 Cognitive, emotional and psychological correlates

Three studies examined the association between a child’s temperament or personality and screen time (Howe, 2017, Downing, 2017, Leppänen, 2020). None of these found an association. In summary, we found no evidence for an association of a child’s temperament or personality with screen time.

### 3.4 Environmental correlates

#### 3.4.1 Physical environmental correlates

Sixteen studies examined physical environmental correlates of screen time, of which twelve studies examined the association between having an electronic device in the child’s (bed)room. Nine of these twelve studies found that having an electronic device in the bedroom was associated with more screen time (Hish, 2021, Carson and Janssen, 2012, Downing, 2017, Lee, 2018, Lee, 2021, Njoroge, 2013, Rodrigues, et al., xxxx, Sijtsma, 2015, Thompson, 2018) whereas three studies found no significant association (Birken, 2011, Bleakley et al., 2013, Vaala and Hornik, 2014). Four out of seven studies found that having an electronic device at home was associated with more screen time (Downing, 2017, Määttä, 2017, Sijtsma, 2015, Vaala and Hornik, 2014) whereas three studies found no significant association (Birken, 2011, Bleakley et al., 2013, Lee, 2021). Four out of five studies examining the association between neighborhood-related factors (e.g., safety and living on a cul-de-sac) and screen time found no significant association (Carson et al., 2014, Downing, 2017, Lee, 2018, Wang, 2020) while one study found a perceived safer neighborhood associated with less screen time (Lee, 2021). Household features (e.g., having a yard, fences) were associated with less screen time in two studies (Downing, 2017, Morowatisharifabad et al., 2015). Inconsistent results were found for the association between the availability of toys (Downing, 2017, Vaala and Hornik, 2014) and screen time.

In summary, we found moderate evidence for having an electronic device in the (bed)room where the child sleeps and household features being associated with screen time. We found no evidence for an association of neighborhood-related factors with screen time, and inconsistent evidence for an association of having electronic devices at home and the availability of toys with screen time.

#### 3.4.2 Economic correlates

Twenty-nine studies examined economic correlates with household income and parental education (both n = 16 studies) mostly examined. Eight out of 16 studies found a negative association of household income with screen time (Brown, 2010, Carson and Janssen, 2012, Carson and Kuzik, 2017, Downing, 2017, Määttä, 2017, Nikken and Schols, 2015, Tombeau Cost, 2020, Waller, 2021) while another eight found no significant association (Bernard, 2017, Hish, 2021, Krogh, et al., xxxx, Matarma, 2016, Birken, 2011, Bleakley et al., 2013, Njoroge, 2013, Thompson and Christakis, 2007). Ten studies found a negative association between parental education and screen time (Bernard, 2017, Detnakarintra, 2020, Krogh, et al., xxxx, Matarma, 2016, Xu, 2016, Bleakley et al., 2013, Carson et al., 2014, Määttä, 2017, Nikken and Schols, 2015, Waller, 2021) and six studies found no significant association (Hish, 2021, Álvarez, et al., xxxx, Carson and Kuzik, 2017, Downing, 2017, Morowatisharifabad et al., 2015, Thompson and Christakis, 2007). Three out of seven studies examining parental employment found an association between (full-time) maternal employment and more screen time (Matarma, 2016, Birken, 2011, Bleakley et al., 2013), one study found maternal unemployment associated with more screen time (Vaala and Hornik, 2014), two studies found being (part-time) employed associated with less screen time (Xu, 2016, Brown, 2010), and two studies found no significant association (Downing, 2017, Kim, et al., xxxx). None of the six studies examining parental marital status/living together (Bernard, 2017, Álvarez, et al., xxxx, Carson and Janssen, 2012, Carson and Kuzik, 2017, Downing, 2017, Tombeau Cost, 2020) or of the four studies examining socio-economic level indicators (e.g., measures of position, levels of area deprivation) (Howe, 2017, Álvarez, et al., xxxx, Barr, 2010, Rodrigues, 2020) found a significant association with screen time. One study found that living in a rural area was associated with less screen time (Contreras, et al., xxxx) while another study found that living in an urban area was associated with less screen time (Wang, 2020).

In summary, we found no evidence for an association of parental marital status/living together or socio-economic level indicators with screen time. We found inconsistent evidence for an association of household income, parental education, parental employment, and living area with screen time.

#### 3.4.3 Sociocultural correlates

Forty-two studies examined sociocultural correlates of screen time. When examining parental socio-demographic correlates of screen time, none of the included studies found an association between parental gender and screen time (Carson and Kuzik, 2017, Nikken and Schols, 2015). For parental age, five out of six studies found no significant association (Bernard, 2017, Howe, 2017, Krogh, et al., xxxx, Carson and Kuzik, 2017, Downing, 2017). Two studies examined the country in which families lived and screen time (Álvarez, et al., xxxx, De Craemer, 2015). One study found that children in Chile had more screen time compared to children in Colombia and Spain (Álvarez, et al., xxxx), while another study found that children from Germany had higher screen time compared to children from Spain, Belgium, Poland, Bulgaria and Greece (De Craemer, 2015).

Seventeen out of 20 studies found a positive association of parental screen time associated with children’s screen time (Barber, 2017, Howe, 2017, Matarma, 2016, Xu, 2016, Birken, 2011, Bleakley et al., 2013, Carson and Janssen, 2012, Carson et al., 2020, De Decker, 2015, Downing, 2017, Lee, 2018, Lee, 2021, Määttä, 2017, Morowatisharifabad et al., 2015, Nikken and Schols, 2015, Vaala and Hornik, 2014, Wiseman et al., 2019), and three studies found no significant associations (Bernard, 2017, Abbott, 2016, Tang, 2018). Nine of the 14 studies found that having rules around screen time (e.g., regarding duration or content) was associated with less screen time (Thompson, 2015, Xu, 2016, Birken, 2011, De Decker, 2015, Lee, 2018, Sanders, 2016, Tang, 2018, Thompson, 2018, Wiseman et al., 2019). Four studies found no significant association (Barr, 2010, Bleakley et al., 2013, Lee, 2021, Wang, 2020) while one study found a positive association (Määttä, 2017). Five studies found that being in childcare compared to being at home was associated with less screen time (Hish, 2021, Matarma, 2016, Xu, 2016, Carson and Kuzik, 2017, Vaala and Hornik, 2014) whereas two studies found no significant association (Carson et al., 2014, Njoroge, 2013). Two out of four studies found that having the TV on during meals or snacks was associated with more screen time (Xu, 2016, Tang, 2018), while two studies found no significant association (Birken, 2011, Thompson, 2018). Having a TV on at home (Barber, 2017, Xu, 2016, Wiseman et al., 2019) or parents finding a higher amount of TV time suitable (descriptive norm) (Carson and Janssen, 2012, Määttä, 2017, Vaala and Hornik, 2014) were both associated with more screen time in all included studies examining this correlate. Monitoring screen time was associated with lower screen time in two studies (Tang, 2018, Wiseman et al., 2019).

Two studies found no significant association between the level of parental physical activity and children’s screen time (Howe, 2017, Downing, 2017). When examining parenting styles, children of authoritarian mothers or partners or permissive mothers had more screen time in one study (Howe, 2017), while nurturing authoritative parenting was associated with less screen time in another study (Detnakarintra, 2020). Other associations with inconsistent results among two studies were between parental/adult support for physical activity (Downing, 2017, Wiseman et al., 2019), turning on the TV to control behavior (Tang, 2018, Wiseman et al., 2019), the number of visits to the park or active places (Downing, 2017, French, 2017), parent–child co-TV-watching (Birken, 2011, Bleakley et al., 2013) or parent–child interactions (Detnakarintra, 2020, Morowatisharifabad et al., 2015) and screen time.

Parental attitudes were examined in nine studies of which six found a less concerned attitude around screen time associated with higher screen time (Barber, 2017, Carson and Janssen, 2012, De Decker, 2015, Määttä, 2017, Vaala and Hornik, 2014, Wang, 2020) and three studies found no significant association (Hinkley, 2017, Nikken and Schols, 2015, Sanders, 2016). Parents placing high value and importance on physical activity was associated with less screen time in two studies (Downing, 2017, Wiseman et al., 2019).

Twelve studies examined parental (mental or physical) health as a correlate of screen time. Five studies examined parental weight status of which four studies found no significant association between parental weight status and screen time (Bernard, 2017, Howe, 2017, Matarma, 2016, Downing, 2017) and one study found a positive association (Brown, 2010). Three out of four studies found no significant association between parental depression and screen time (Bleakley et al., 2013, Cowderoy, 2020, Kim, et al., xxxx) and one study found more depressive symptoms associated with more screen time (Morrissey, 2014). Two studies examining parental stress found higher levels of parental stress associated with more screen time (Thompson and Christakis, 2007, Tombeau Cost, 2020) whereas two studies found no significant association (Barber, 2017, Kim, et al., xxxx). Parental wellbeing (including having a physical disability) was examined in two studies, which both demonstrated no significant association with screen time (Bleakley et al., 2013, Downing, 2017).

Six out of seven studies found a higher parental self-efficacy to limit screen time associated with lower screen time (Hnatiuk, 2015, Carson and Janssen, 2012, Downing, 2017, Lee, 2021, Määttä, 2017, Sanders, 2016) while one study found no significant association (Lee, 2018). Two studies found no significant association between positive outcome expectations from screen time and screen time (Lee, 2018, Lee, 2021). When examining negative outcome expectations from screen time, one study found a positive association (Lee, 2021) while another study found no significant association (Lee, 2018). One study found higher parental confidence associated with less screen time (Njoroge, 2013) while another study found no significant association (Vaala and Hornik, 2014).

In summary, we found moderate evidence for an association of country, parental screen time, having a TV on at home, descriptive norms, placing high value and importance on physical activity, monitoring screen time, being away from home care and parental self-efficacy with screen time. We found no evidence for an association of parental sex, age, physical activity, weight status, depression, wellbeing, and positive outcome expectations with screen time. We found inconsistent evidence for an association of having rules around screen time, having the TV on during meals/snacks or to control behavior, parental support for physical activity, park or active place visits, co-watching, parent–child interactions, parenting styles, parental attitudes, concerns, mental or physical health, negative outcome expectations and confidence to limit TV time with screen time.

## 4 Discussion

### 4.1 Overview of findings

This systematic review aimed to summarize the evidence on correlates of screen time in children aged 0–5 years. For a number of correlates we found moderate or no evidence for associations with screen time, however, we were unable to draw strong conclusions as for the majority of correlates we found inconsistent or insufficient evidence.

The methodological quality of included studies was rated as moderate or low for almost 95% of studies, with the majority of studies having a low methodological quality. Only two studies had a high methodological quality. Over half of the assessed studies used questionnaires that are not or have unknown validity and/or reliability to assess screen time in this young age group. There was a large variation in type of screen time measured (e.g., total screen time, TV time, hand-held devices). Many types of screen time (e.g. hand-held devices, electronic media use) were only studied in one to three studies, and only TV time and total screen time were studied sufficiently to report correlates separately. As correlates might differ for different types of screen time, future studies should explore various types of screen time, including using smartphones and tablets. The need to report and/or improve psychometric properties of screen time assessments was recently highlighted in a systematic review examining assessment in early childhood (Byrne et al., 2021, Arts, 2022). Additionally, studies scored low on the items ‘selection bias’ and ‘participation rate’. For selection bias, the low scores are due to a combination of non-representative samples and lack of information on recruitment rates. For participation rate, this was mostly due to a lack of information. Additional information provided through references did not yield the required information. Finally, most studies had a cross-sectional design where as a longitudinal design provides stronger evidence.

Over 55% of the included studies were published in the last five years, confirming screen time in early childhood is a quickly developing area of research and the pertinence of our review. Overall, our results are in line with previous reviews (Hinkley, 2010, Hoyos Cillero and Jago, 2010, Duch, 2013). There is inconsistent or insufficient evidence of a large number of examined correlates, highlighting the need for more research in this area. Our results provided new evidence for correlates that were absent or undetermined in previous reviews (e.g., child’s sleep duration and parental age) as well as confirmed results from previous reviews (e.g., child sex and parental employment).

The inconsistent or insufficient evidence (70% of examined correlates) might be explained by the large variation of correlates examined as well as the measures used to assess them. When possible, studies examining a similar construct but using different measures were therefore combined. Compared to previous reviews, studies included in our review focused more often on the different types of screen time, including handheld devices in addition to TV viewing (Vanderloo, 2014, Xu et al., 2015, Duch, 2013).

Most examined correlates were related to parents, underscoring the important role parents play in young children’s lives through role modelling and creating a home environment (Rhee, 2008). This reviews builds on previous reviews when examining parental correlates by adding to the evidence-based in this area of research for several correlates (e.g., monitoring screen time and parental physical activity). Even though we found inconsistent or insufficient evidence for most included correlates, we were able to draw conclusions on 15 sociocultural correlates. Results were in line with previous reviews for parental screen time (Hoyos Cillero and Jago, 2010, Xu et al., 2015) and self-efficacy (Xu et al., 2015). We found different results compared to Hoyos et al. (2010) for parental BMI, parental depressive symptoms (no evidence in this review compared to a positive association in previous review) and rules around TV use (insufficient evidence in this review compared to a negative association in previous review). These differences can be explained by different inclusion criteria: Hoyos et al. (2010) included studies with a mean child’s age up to 7 years and studies using adherence to guidelines (yes/no) as an assessment of screen time (Hoyos Cillero and Jago, 2010). The moderate association found between country and screen time are likely caused by underlying differences in cultural, socio-economic or other factors, and need further investigation.

### 4.2 Strengths and Limitations

Strengths of this review are the examination of correlates per type of screen time (although there was insufficient evidence to present the results per type), the methodological quality assessment, the best evidence synthesis and the inclusion of a large number of correlates and the full early childhood age range (0–5 years).

As there was a large variation in how correlates were defined and measured, we combined studies examining a similar construct, which might have led to more inconsistent results. Additional limitations are not contacting authors of the original studies for more information, due to practical reasons, and only including English publications. Furthermore, we could not evaluate potential publication bias or selective reporting of significant findings, which could have influenced our results.

### 4.3 Recommendations for future research

To further examine correlates of screen time in early childhood and to inform future interventions we recommend:

Focusing on sociocultural correlates, especially parental factors, physical environmental factors, behavioral factors and economic factors, as there are various correlates with undetermined evidence in these categories.

Investing in better methodologies and improved reporting to ensure high quality studies, including: conducting longitudinal studies; the use of valid and reliable assessment of different types of screen time (e.g., smartphone use, television viewing) and potential correlates; recruiting more representative samples; reporting of study details, especially around recruitment and participation rates.

Exploring interactions between factors that could influence screen time, as currently few studies examined such interactions (e.g. gender differences, ethnicity and culture).

## 5 Conclusions

This systematic review summarized the correlates of screen time in typically developing children aged 0–5 years. Based on these results, we recommend future interventions to focus on factors related to the physical environment (e.g., the presence of electronic devices in the bedroom) as well as on sociocultural factors (e.g., parental self-efficacy). However, the evidence for most included correlates was inconsistent or insufficient and as such more high-quality research is needed to inform future interventions.

## 6 Declarations

Ethics approval and consent to participate: Ethics approval was not needed as this study was based on publicly available anonymized data.

## Funding

This study was funded by the Netherlands Organization for Health Research and Development (ZonMw; Project No. 546003008) and the Bernard van Leer Foundation. The funding bodies had no role in the design of the study; the collection, analysis, and interpretation of data; or the writing of the manuscript.

## Declaration of Competing Interest

The authors declare that they have no known competing financial interests or personal relationships that could have appeared to influence the work reported in this paper.

## Data Availability

No data was used for the research described in the article.
