# Supplementary material for: Correlates of screen time in the early years (0–5 years): A systematic review
Source: Prev Med Rep. 2023 Apr 19;33:102214. doi: 10.1016/j.pmedr.2023.102214 (PMC10201873; doi:10.1016/j.pmedr.2023.102214)
Supplement: Supplementary data 1 [file mmc1.docx]

**Supplementary File 1. Overview of search terms**

Pubmed

(((regression[tiab] OR predicted[tiab] OR relation[tiab] OR relationship*[tiab] OR related[tiab] OR associated[tiab] OR association*[tiab] OR correlat*[ti] OR determinant*[ti] OR risk factor*[ti] OR correlat*[ot] OR determinant*[ot] OR risk factor*[ot]) AND ("Infant"[Mesh:noexp] OR "Infant, Newborn"[Mesh:noexp] OR "Child, Preschool"[Mesh] OR infan*[tiab] OR newborn*[tiab] OR new-born*[tiab] OR neonate*[tiab] OR baby[tiab] OR babies[tiab] OR toddler*[tiab] OR preschool*[tiab] OR pre-school*[tiab] OR kindergarten*[tiab] OR childcare*[tiab] OR daycare*[tiab] OR nurser*[tiab] OR ECEC[tiab] OR early childhood[tiab] OR early years[tiab] OR early life[tiab] OR children[tiab]) AND (screen time[Mesh:NoExp] OR screen time[tiab] OR screen entertainment[tiab] OR screen based[tiab] OR screen viewing[tiab] OR computer time[tiab] OR computer use[tiab] OR computer game[tiab] OR TV[tiab] OR television[tiab] OR video[tiab] OR videogam*[tiab] OR gaming[tiab] OR tablet use[tiab] OR tablet time[tiab] OR iPad[tiab] OR smartphone[tiab] OR iPhone[tiab] OR mobile phone[tiab])))) NOT ("Diseases Category"[Majr] OR "Attention Deficit and Disruptive Behavior Disorders"[Majr] OR "Child Development Disorders, Pervasive"[Majr])) NOT ("Animals"[Mesh] NOT "Humans"[Mesh])

Embase

((((regression:ab,ti,kw OR predicted:ab,ti,kw OR relation:ab,ti,kw OR relationship*:ab,ti,kw OR related:ab,ti,kw OR associated:ab,ti,kw OR association*:ab,ti,kw OR correlat*:ti OR determinant*:ti OR 'risk factor*':ti OR correlat*:kw OR determinant*:kw OR 'risk factor*':kw) AND ('infant'/de OR 'baby'/exp OR 'newborn'/exp OR 'preschool child'/exp OR 'toddler'/exp OR 'early childhood'/exp OR 'early life'/exp OR infan*:ab,ti,kw OR newborn*:ab,ti,kw OR 'new-born*':ab,ti,kw OR neonate*:ab,ti,kw OR baby:ab,ti,kw OR babies:ab,ti,kw OR toddler*:ab,ti,kw OR preschool*:ab,ti,kw OR 'pre-school*':ab,ti,kw OR kindergarten*:ab,ti,kw OR childcare*:ab,ti,kw OR daycare*:ab,ti,kw OR nurser*:ab,ti,kw OR ECEC:ab,ti,kw OR 'early childhood':ab,ti,kw OR 'early years':ab,ti,kw OR 'early life':ab,ti,kw OR children:ab,ti,kw) AND ('screen time'/exp OR 'screen time':ab,ti,kw OR 'screen entertainment':ab,ti,kw OR 'screen based':ab,ti,kw OR 'screen viewing':ab,ti,kw OR 'computer time':ab,ti,kw OR 'computer use':ab,ti,kw OR 'computer game':ab,ti,kw OR 'TV time':ab,ti,kw OR 'television':ab,ti,kw OR 'video':ab,ti,kw OR 'videogam*':ab,ti,kw OR 'gaming':ab,ti,kw OR 'tablet use':ab,ti,kw OR 'tablet time':ab,ti,kw OR 'iPad':ab,ti,kw OR 'smartphone':ab,ti,kw OR 'iPhone':ab,ti,kw OR 'mobile phone':ab,ti,kw)) NOT ('diseases'/exp/mj OR 'attention deficit disorder'/exp/mj OR 'autism'/exp/mj)) NOT ([animals]/lim NOT [humans]/lim)) NOT ('conference abstract'/it OR 'conference review'/it)

SPORTDiscus

(((TI (regression OR predicted OR relation OR relationship* OR related OR associated OR association* OR correlat* OR determinant* OR "risk factor*") OR AB (regression OR predicted OR relation OR relationship* OR related OR associated OR association*) OR KW (regression OR predicted OR relation OR relationship* OR related OR associated OR association* OR correlat* OR determinant* OR "risk factor*")) AND (TI (infan* OR newborn* OR "new-born*" OR neonate* OR baby OR babies OR toddler* OR preschool* OR "pre-school*" OR kindergarten* OR childcare* OR daycare* OR nurser* OR ECEC OR "early childhood" OR "early years" OR "early life" OR children) OR AB (infan* OR newborn* OR "new-born*" OR neonate* OR baby OR babies OR toddler* OR preschool* OR "pre-school*" OR kindergarten* OR childcare* OR daycare* OR nurser* OR ECEC OR "early childhood" OR "early years" OR "early life" OR children) OR KW (infan* OR newborn* OR "new-born*" OR neonate* OR baby OR babies OR toddler* OR preschool* OR "pre-school*" OR kindergarten* OR childcare* OR daycare* OR nurser* OR ECEC OR "early childhood" OR "early years" OR "early life" OR children)) AND ("screen time" OR "screen entertainment" OR "screen based" OR "screen viewing" OR "computer time" OR "computer use" OR "computer game" OR "TV" OR television OR OR "video" OR videogam* OR "gaming" OR "tablet use" OR "tablet time" OR "iPad" OR "smartphone" OR "iPhone" OR "mobile phone") OR AB("screen time" OR "screen entertainment" OR "screen based" OR "screen viewing" OR "computer time" OR "computer use" OR "computer game" OR "TV" OR television OR OR "video" OR videogam* OR "gaming" OR "tablet use" OR "tablet time" OR "iPad" OR "smartphone" OR "iPhone" OR "mobile phone") OR KW("screen time" OR "screen entertainment" OR "screen based" OR "screen viewing" OR "computer time" OR "computer use" OR "computer game" OR "TV" OR television OR OR "video" OR videogam* OR "gaming" OR "tablet use" OR "tablet time" OR "iPad" OR "smartphone" OR "iPhone" OR "mobile phone") NOT ((MM "CEREBRAL palsy" OR MM "ATTENTION-deficit hyperactivity disorder" OR MM "ATTENTION-deficit-disordered children" OR TI ("cerebral palsy" OR autism OR autistic OR "attention deficit" OR ADHD) OR KW ("cerebral palsy" OR autism OR autistic OR "attention deficit" OR ADHD))))
