# Supplementary material for: Correlates of screen time in the early years (0–5 years): A systematic review
Source: Prev Med Rep. 2023 Apr 19;33:102214. doi: 10.1016/j.pmedr.2023.102214 (PMC10201873; doi:10.1016/j.pmedr.2023.102214)
Supplement: Supplementary data 2 [file mmc2.docx]

**Supplementary File 2. Quality Assessment Tool for Quantitative Studies’ (EPHPP)**

**A. SELECTION BIAS**

| Q1 Representative | Very likely^1^ | Very/Somewhat likely^2^ | Not likely^3^/Not described |
| --- | --- | --- | --- |
| Q2 Participation | 80-100% | 80-100%/60-79% | Less than 60%/can’t tell |
| RATING | GOOD | FAIR | POOR |

^1^ Random individuals selected from target population.
^2^ Referred systematically from schools/ECEC or health settings.

^3^ Self-referred

**B. STUDY DESIGN**

| Design | Longitudinal design/RCT^1^ | RCT^1^/Controlled clinical trial/Cross-sectional | Other/can’t tell |
| --- | --- | --- | --- |
| Randomized | NA/YES | NA/NO | NA/No |
| Described as randomized | NA/YES | NA/NO | NA/No |
| RATING | GOOD | FAIR | Poor |

^1^ Randomized controlled trial.

**C. CONFOUNDERS**

| Controlled for confounders | Controlled for pre-intervention/baseline scores, sex and age | Controlled for pre-intervention/baseline scores, sex or age | Not (sufficiently) controlled for pre-confounders |
| --- | --- | --- | --- |
| RATING | GOOD | FAIR | POOR |

**D. BLINDING**

| Q1 Assessors | YES |  | NO / can’t tell | NA |
| --- | --- | --- | --- | --- |
| RATING | GOOD | FAIR | POOR | NA |

**E. DATA COLLECTION METHODS**

| Q1 Exposure: valid | Objective or validity >0.7: YES/NO/Can’t tell | At least three times a YES score | Two times a YES score | No or one YES score |
| --- | --- | --- | --- | --- |
| Q2 Exposure: reliable | Objective or reliability >0.7: YES/NO/Can’t tell |  |  |  |
| Q3 Outcome: valid | Validity >0.7: YES/NO |  |  |  |
| Q4 Outcome: reliable | Reliability >0.7: YES/NO/Can’t tell |  |  |  |
| RATING |  | GOOD | FAIR | POOR |

**F1. PARTICIPATION RATE**

| Q1 Reported at baseline | YES | YES | YES/NO | NA |
| --- | --- | --- | --- | --- |
| Q2 Completion at baseline | 80-100% | 60-79% | <60% / can’t tell | NA |
| RATING | GOOD | FAIR | POOR | NA |

**F2. WITHDRAWALS AND DROP-OUTS**

| Q1 Reported | YES | YES | YES/NO | NA |
| --- | --- | --- | --- | --- |
| Q2 Completion | 80-100% | 60-79% | <60% / can’t tell | NA |
| RATING | GOOD | FAIR | POOR | NA |

**G. INTERVENTION INTEGRITY**

| Q1 Intervention delivery | 80-100% | 80-100%/60-79% | <60% / can’t tell | NA |
| --- | --- | --- | --- | --- |
| Q2 Consistency measured | YES | YES/NO | NO/Can’t tell | NA |
| Q3 Contamination between groups | NO | NO | YES/Can’t tell | NA |
| RATING | GOOD | FAIR | POOR | NA |

**H. ANALYSES**

| Q1 Allocation / analysis unit^5^ | Appropriate | Appropriate | Not appropriate / can’t tell |
| --- | --- | --- | --- |
| Q2 Subjects = 10x variables | YES | No | NO/can’t tell |
| Q3 Analyses appropriate^6^ | YES | YES | NO/can’t tell |
| Q4 Intention to treat^7^ | YES/NA | YES/can’t tell/NA | NO/can’t tell/NA |
| RATING | GOOD | FAIR | POOR |

^5^ Level of allocation and analysis should be the same for a strong analysis (multi-level) if required

^6^ Effect sizes reported, i.e. beta.

^7^ Only applicable for longitudinal studies

**OVERALL METHODOLOGICAL QUALITY SCORE**

**HIGH:** NO POOR SCORES

**MODERATE:** MAX. ONE POOR SCORE

**WEAK:** TWO OR MORE POOR SCORES

**References**

Jackson, N. and E. Waters (2005). "Criteria for the systematic review of health promotion and public health interventions." Health Promot Int 20(4): 367-374.

Thomas, B. H., et al. (2004). "A process for systematically reviewing the literature: providing the research evidence for public health nursing interventions." Worldviews Evid Based Nurs 1(3): 176-184.
