# Supplementary material for: Correlates of screen time in the early years (0–5 years): A systematic review
Source: Prev Med Rep. 2023 Apr 19;33:102214. doi: 10.1016/j.pmedr.2023.102214 (PMC10201873; doi:10.1016/j.pmedr.2023.102214)
Supplement: Supplementary data 3 [file mmc3.docx]

Supplementary file 3. Summary of results regarding correlates of screen time in young children (<4 years), separate for total screen time (3.1) and TV time (3.2).

3.1: Summary of results regarding correlates of total screen time in young children (<4 years).

| **Correlate** | **Included studies^a, b^** | **Summary coding**  **(n/N, (%))^a,b^** | **Evidence synthesis** |
| --- | --- | --- | --- |
| ***Biological correlates*** | | | |
| Sex | + + **0** 0 0 0 | ? | Based on inconsistent findings among studies with low-to-high methodological quality, there is insufficient evidence for an association between sex and screen time. |
| Age | + + + 0 0 0 | ? | Based on inconsistent findings among studies with low-to-moderate methodological quality, there is insufficient evidence for an association between age and screen time. |
| Ethnicity/race child or parent/country of birth parent | + + + 0 - - | ? | Based on inconsistent findings among studies with low-to-moderate methodological quality, there is insufficient evidence for an association between ethnicity/race of the child or parent and screen time. |
| Siblings/nr of children in the house | 0 0 0 | 0 (3/3, 100%) | Based on consistent findings among studies with low-to-moderate methodological quality, there is no significant evidence for an association between having siblings/the number of children in the house and screen time. |
| First-born | 0 0 0 | 0 (3/3, 100%) | Based on consistent findings among studies with low-to-moderate methodological quality, there is no significant evidence for an association between being first-born and screen time. |
| BMI | 0 - | ? | Based on inconsistent findings among studies with low-to-moderate methodological quality, there is insufficient evidence for an association between BMI and screen time. |
| ***Behavioral attributes and skills-related correlates*** | | | |
| Sleep duration | - - | - (2/2, 100%) | Based on consistent findings among studies with low-to-moderate methodological quality, there is moderate evidence for a negative association between sleep duration and screen time. |
| ***Cognitive, emotional or psychological correlates*** | | | |
| Temperament/personality | 0 0 | 0 (2/2, 100%) | Based on consistent findings among studies with low-moderate methodological quality, there is in no significant evidence for an association between temperament/personality and screen time. |
| ***Physical environmental correlates*** | | | |
| Electronic devices/computer/ TV in (bed)room where child sleeps | + + + + + 0 | + (5/6, 83%) | Based on consistent findings among studies with low-to-moderate methodological quality, there is moderate evidence for a positive association between having electronic devices in the (bed) room where the child sleeps and screen time. |
| Electronic devices/screen-based/TVs at home | + + + 0 0 | ? | Based on inconsistent findings among studies with low-to-moderate methodological quality, there is insufficient evidence for an association between having electronic devices at home and screen time. |
| Neighbourhood-related factors | 0 0 0 0 - | 0 (4/5, 80%) | Based on consistent findings among studies with low-to-moderate quality methodological quality, there is no significant evidence for an association between neighbourhood-related factors and screen time. |
| ***Economic correlates*** | | | |
| Parental education | 0 0 0 0 0 0 - - - - - - - | ? | Based on inconsistent findings among studies with low-to-moderate methodological quality, there is insufficient evidence for an association between parental education and screen time. |
| Family income | 0 0 0 0 0 - - - - - | ? | Based on inconsistent findings among studies with low-to-moderate methodological quality, there is insufficient evidence for an association between family income and screen time. |
| Parental employment | + + - | ? | Based on inconsistent findings among studies with low-to-moderate methodological quality, there is insufficient evidence for an association between parental employment and screen time. |
| Marital status/parents live together | 0 0 0 0 0 | 0 (5/5, 100%) | Based on consistent findings among studies with low-to-moderate methodological quality, there is no significant evidence for an association between marital status/parents living together and screen time. |
| ***Sociocultural correlates*** | | | |
| Parental screen time/media use/modelling | + + + + + + + + + 0 0 | + (9/11, 82%) | Based on consistent findings among studies with low-to-moderate methodological quality, there is moderate evidence for a positive association between parental screen time and children’s screen time. |
| Rules around screen time | + 0 0 - - - - - - | ? | Based on inconsistent findings among studies with low-to-moderate methodological quality, there is insufficient evidence for an association between having rules around screen time and screen time. |
| TV on at home | + + | + (2/2, 100%) | Based on consistent findings among studies with low-to-moderate methodological quality, there is moderate evidence for a positive association between having a TV at home and screen time. |
| Monitoring screen time | - - | - (2/2, 100%) | Based on consistent findings among studies with low-to-moderate methodological quality, there is moderate evidence for a negative association between monitoring screen time and children’s screen time. |
| Away from home care | 0 - - - | ? | Based on inconsistent findings among studies with low-to-moderate methodological quality, there is insufficient evidence for a negative association between being away from home care and screen time. |
| Placing high importance and value on physical activity | - - | - (2/2, 100%) | Based on consistent findings among studies with low-to-moderate methodological quality, there is moderate evidence for a negative association between placing high importance and value on physical activity and screen time. |
| TV on during meals/snacks | + + 0 | ? | Based on inconsistent findings among studies with low-to-moderate methodological quality, there is insufficient evidence for an association between having a TV on during meals and screen time. |
| Parental weight status | 0 0 0 | 0 (3/3, 100%) | Based on consistent findings among studies with low-to-moderate methodological quality, there is no significant evidence for an association between parental weight status and screen time. |
| TV on to control behaviour | + 0 | ? | Based on inconsistent findings among studies with low-to-moderate methodological quality, there is insufficient evidence for an association between having a TV on to control behavior and screen time. |
| Support/reinforcement for physical activity from other adults | + - | ? | Based on inconsistent findings among studies with low-to-moderate methodological quality, there is insufficient evidence for an association between support for physical activity from other adults and screen time. |
| Positive outcome expectations | 0 0 | 0 (2/2, 100%) | Based on consistent findings among studies with low-to-moderate methodological quality, there is no significant evidence for an association between between positive outcome expectations and screen time. |
| Parental depression | + 0 | ? | Based on inconsistent findings among studies with low-to-moderate methodological quality, there is insufficient evidence for an association between parental depression and screen time. |
| Parental mental health: stress | + + | + (2/2, 100%) | Based on consistent findings among studies with low-to-moderate methodological quality, there is moderate evidence for a positive association between parental mental health and screen time. |
| Parental age | + 0 0 0 0 | 0 (4/5,80%) | Based on consistent findings among studies with low-to-moderate methodological quality, there is no significant evidence for an association between parental age and screen time. |
| Parental attitudes | + + 0 0 | ? | Based on inconsistent findings among studies with low-to-moderate methodological quality, there is insufficient evidence for an association between parental attitudes and screen time. |
| Negative outcome expectations | + 0 | ? | Based on inconsistent findings among studies with low-to-moderate methodological quality, there is insufficient evidence for an association between negative outcome expectations and screen time. |
| Parental self-efficacy | 0 - - - - | - (6/7,86%) | Based on consistent findings among studies with low-to-moderate methodological quality, there is moderate evidence for a negative association between parental self-efficacy and screen time. |

^a^ bold indicates results from high quality study; ^b^ summary score: + = consistent positive association, - = consistent negative association, 0 = consistent no association, ? = inconsistent findings; score is based on all studies unless 2 or more high quality studies were available. Abbreviations: BMI = body mass index, PA = physical activity, TV = television.

.

3.2: Summary of results regarding correlates of TV time in young children (<4 years).

| **Correlate** | **Included studies^a, b^** | **Summary coding**  **(n/N, (%))^a,b^** | **Evidence synthesis** |
| --- | --- | --- | --- |
| ***Biological correlates*** | | | |
| Sex | + **0** 0 0 0 0 0 0 0 0 0 - | 0 (10/12, 83%) | Based on consistent findings among studies with low-to-high methodological quality, there is no significant evidence for an association between sex and screen time. |
| Age | **+** + + + + + + 0 0 | + (7/9, 78%) | Based on consistent findings among studies with low-to-high methodological quality, there is moderate evidence for a positive association between age and screen time. |
| Ethnicity/race child or parent/country of birth parent | + + + + 0 0 0 0 **-** | ? | Based on inconsistent findings among studies with low-to-high methodological quality, there is insufficient evidence for an association between ethnicity/race of the child or parent and screen time. |
| Siblings/nr of children in the house | 0 0 0 0 0 - | 0 (5/6, 83%) | Based on consistent findings among studies with low-to-moderate methodological quality, there is no significant evidence for an association between having siblings/the number of children in the house and screen time. |
| First-born | + 0 0 | ? | Based on inconsistent findings among studies with low-to-moderate methodological quality, there is insufficient evidence for an association between being first-born and screen time. |
| BMI | 0 0 | 0 (2/2, 100%) | Based on consistent findings among studies with low-to-moderate methodological quality, there is no significant evidence for an association between BMI and screen time. |
| ***Physical environmental correlates*** | | | |
| Electronic devices/computer/ TV in (bed)room where child sleeps | + + + + 0 0 | ? | Based on inconsistent findings among studies with low-to-moderate methodological quality, there is insufficient evidence for an association between having electronic devices in the (bed) room where the child sleeps and screen time. |
| Electronic devices/screen-based/TVs at home | + 0 | ? | Based on inconsistent findings among studies with low-to-moderate methodological quality, there is insufficient evidence for an association between having electronic devices at home and screen time. |
| ***Economic correlates*** | | | |
| Parental education | 0 0 0 0 - - - | ? | Based on inconsistent findings among studies with low-to-moderate methodological quality, there is insufficient evidence for an association between parental education and screen time. |
| Family income | 0 0 0 0 0 - - - | ? | Based on inconsistent findings among studies with low-to-moderate methodological quality, there is insufficient evidence for an association between family income and screen time. |
| Socio-economic variables | 0 0 0 0 | 0 (4/4, 100%) | Based on consistent findings among studies with low-to-moderate methodological quality, there is no significant evidence for an association between socio-economic variables and screen time. |
| Parental employment | + + 0 - | ? | Based on inconsistent findings among studies with low-to-moderate methodological quality, there is insufficient evidence for an association between parental employment and screen time. |
| Marital status/parents live together | 0 0 0 | 0 (3/3, 100%) | Based on consistent findings among studies with low-to-moderate methodological quality, there is no significant evidence for an association between marital status/parents living together and screen time. |
| ***Sociocultural correlates*** | | | |
| Parental screen time/media use/modelling | + + + + + + + + + 0 | + (9/10, 90%) | Based on consistent findings among studies with low-to-moderate methodological quality, there is moderate evidence for a positive association between parental screen time and children’s screen time. |
| Rules around screen time | 0 0 - - - | ? | Based on inconsistent findings among studies with low-to-moderate methodological quality, there is insufficient evidence for an association between having rules around screen time and screen time. |
| Away from home care | 0 - - - | - (3/4, 75%) | Based on consistent findings among studies with low-to-moderate methodological quality, there is moderate evidence for a negative association between being away from home care and screen time. |
| Descriptive norms | + + | + (2/2, 100%) | Based on consistent findings among studies with low-to-moderate methodological quality, there is moderate evidence for a positive association between descriptive norms and screen time. |
| Parental weight status | + 0 | ? | Based on consistent findings among studies with low-to-moderate methodological quality, there is no significant evidence for an association between parental weight status and screen time. |
| Parental sex | 0 0 | 0 (2/2, 100%) | Based on consistent findings among studies with low-to-moderate methodological quality, there is no significant evidence for an association between parental sex and screen time. |
| Parental depression | 0 0 | 0 (2/2, 100%) | Based on consistent findings among studies with low-to-moderate methodological quality, there is no significant evidence for an association between parental depression and screen time. |
| Parental mental health: stress | 0 0 | 0 (2/2, 100%) | Based on inconsistent findings among studies with low-to-moderate methodological quality, there is insufficient evidence for an association between parental mental health and screen time. |
| Parental age | 0 0 0 | 0 (3/3,100%) | Based on consistent findings among studies with low-to-moderate methodological quality, there is no significant evidence for an association between parental age and screen time. |
| Parental attitudes | + + + + 0 | + (4/5, 80%) | Based on inconsistent findings among studies with low-to-moderate methodological quality, there is insufficient evidence for an association between parental attitudes and screen time. |
| Confidence to limit TV time | 0 - | ? | Based on inconsistent findings among studies with low-to-moderate methodological quality, there is insufficient evidence for an association between the confidence to limit TV time and screen time. |
| Parental self-efficacy | - - | - (2/2, 100%) | Based on consistent findings among studies with low-to-moderate methodological quality, there is moderate evidence for a negative association between parental self-efficacy and screen time. |
| Country | + + | + (2/2, 100%) | Based on consistent findings among studies with low-to-moderate methodological quality, there is moderate evidence for a positive association between countries and screen time. |

^a^ bold indicates results from high quality study; ^b^ summary score: + = consistent positive association, - = consistent negative association, 0 = consistent no association, ? = inconsistent findings; score is based on all studies unless 2 or more high quality studies were available. Abbreviations: BMI = body mass index, PA = physical activity, TV = television.
