# Supplementary material for: Correlates of screen time in the early years (0–5 years): A systematic review
Source: Prev Med Rep. 2023 Apr 19;33:102214. doi: 10.1016/j.pmedr.2023.102214 (PMC10201873; doi:10.1016/j.pmedr.2023.102214)
Supplement: Supplementary data 4 [file mmc4.docx]

Supplementary file 4. Quality Assessment scores of included studies, sorted by methodological quality and author.

| **Study** | **A Selection Bias** | **B Study Design** | **C Confounders** | **D Blinding^1^** | **E Data Collection Methods^2^** | **F1 Participation rate** | **F2 Withdrawals and Drop-outs^3^** | **G Intervention Integrity^1^** | **H Analysis** | **Overall Score** |
| --- | --- | --- | --- | --- | --- | --- | --- | --- | --- | --- |
| Berglind and Tynelius (2017), Sweden | Fair | Fair | Good | NA | Fair | Fair | NA | NA | Fair | **High** |
| Thompson et al. (2010), USA | Fair | Fair | Good | NA | Fair | Fair | NA | NA | Good | **High** |
| Bernard et al. (2017), Singapore | Fair | Good | Good | NA | Fair to poor | Good | Fair | NA | Fair | **High to Moderate** |
| Alvarez et al. (2021), Chile, Colombia and Spain | Poor | Fair | Good | NA | Fair | Good | NA | NA | Good | **Moderate** |
| Birken et al. (2011), Canada | Fair | Fair | Fair | NA | Poor | Good | NA | NA | Good | **Moderate** |
| Contreras et al. (2020), USA | Fair | Fair | Poor | NA | Fair | Good | NA | NA | Fair | **Moderate** |
| De Decker et al. (2015), Australia and Belgium | Fair to poor | Fair | Good | NA | Fair to poor | Good to fair | NA | NA | Good | **Moderate** |
| Flores et al. (2005), USA | Fair | Fair | Fair | NA | Fair | Poor | NA | NA | Good | **Moderate** |
| Krogh et al. (2021), Denmark | Poor | Good | Fair | NA | Fair | Fair | Fair | NA | Good | **Moderate** |
| Maatta et al. (2017), Finland (BMJ) | Poor | Fair | Good | NA | Fair | Good | NA | NA | Good | **Moderate** |
| Thompson and Christakis (2007), USA | Fair | Fair | Good | NA | Poor | Fair | NA | NA | Good | **Moderate** |
| Tombeau et al. (2020), Canada | Poor | Fair | Fair | NA | Fair | Good | NA | NA | Fair | **Moderate** |
| Barber et al. (2017), UK | Good | Good | Fair | NA | Fair to Poor | Poor | Good | NA | Good | **Moderate to low** |
| Carson and Janssen (2012), Canada | Poor | Fair | Good | NA | Fair to Poor | Good | NA | NA | Good | **Moderate to low** |
| Carson et al. (2014), Canada | Poor | Fair | Good | NA | Fair to poor | Fair | NA | NA | Good | **Moderate to low** |
| Downing et al. (2017), Australia | Poor | Fair | Good | NA | Fair to poor | Good | NA | NA | Good | **Moderate to low** |
| Kim et al. (2021), Korea | Poor | Fair | Fair | NA | Fair to poor | Good | NA | NA | Good | **Moderate to low** |
| Lee et al. (2018), Canada | Poor | Fair | Good | NA | Good to poor | Fair | NA | NA | Fair | **Moderate to low** |
| Lee et al. (2020), Canada en Korea | Poor | Fair | Good | NA | Fair to poor | Good | NA | NA | Good | **Moderate to low** |
| Leppanen et al. (2020), Finland | Poor | Fair | Good | NA | Fair to Poor | Good | NA | NA | Fair | **Moderate to low** |
| Nikken and Schols (2015), The Netherlands | Poor | Fair | Good | NA | Fair and poor | Good | NA | NA | Good | **Moderate to low** |
| Njoroge et al. (2013), USA | Poor | Fair | Fair | NA | Fair to poor | Good | NA | NA | Good | **Moderate to low** |
| Tang et al. (2018), Canada | Poor | Fair | Good | NA | Fair or poor | Good | NA | NA | Good | **Moderate to low** |
| Abbott et al. (2015), Australia | Poor | Good | Good | NA | Fair to poor | Poor | Fair | NA | Good | **Low** |
| Barr et al. (2010), USA | Poor | Fair | Good | NA | Good to fair | Poor | NA | NA | Good | **Low** |
| Bleakley et al. (2013), USA | Poor | Fair | Good | NA | Fair to poor | Poor | NA | NA | Good | **Low** |
| Brown et al. (2010), Australia | Poor | Fair | Good | NA | Fair | Poor | NA | NA | Good | **Low** |
| Carson and Kuzik (2017), Canada | Poor | Fair | Good | NA | Good | Poor | NA | NA | Fair | **Low** |
| Carson et al. (2020), Canada | Poor | Fair | Good | NA | Poor | Fair | NA | NA | Good | **Low** |
| Cowderoy et al. (2020), Australia | Poor | Good | Good | NA | Fair (?) | Poor | Good | NA | Fair | **Low** |
| De Craemer et al (2015), Belgium, Bulgaria, Germany, Greece, Poland and Spain | Poor | Fair | Good | NA | Fair | Poor | NA | NA | Good | **Low** |
| Detnakarintra et al. (2020), Thailand | Poor | Good | Good | NA | Fair to poor | Poor | Good | NA | Good | **Low** |
| French et al. (2017), USA | Poor | Fair | Fair | NA | Poor | Poor | NA | NA | Poor | **Low** |
| Hinkley et al. (2017), Australia | Poor | Fair | Good | NA | Poor | Fair | NA | NA | Fair | **Low** |
| Hish et al. (2021), USA | Fair | Good | Good | NA | Fair | Poor | Poor | NA | Fair | **Low** |
| Hnatiuk et al. (2015), Australia | Poor | Good | Fair | NA | Fair | Poor | Poor | NA | Good | **Low** |
| Howe et al. (2017), New Zealand | Poor | Good | Good | NA | Fair to poor | Poor | Fair | NA | Good | **Low** |
| Khan et al. (2017), USA | Poor | Fair | Good | NA | Poor | Poor | NA? | NA | Good | **Low** |
| Maatta et al. (2017), Finland (BMC) | Poor | Fair | Good | NA | Poor | Good | NA | NA | Fair | **Low** |
| Matarma et al. (2016), Finland | Poor | Good | Good | NA | Fair to poor | Good | Poor | NA | Fair | **Low** |
| Morowatisharifa et al. (2015), Iran | Poor | Fair | Poor | NA | Fair | Fair | NA | NA | Fair | **Low** |
| Morrissey et al. (2014), USA | Poor | Good | Good | NA | Poor | Poor | Poor | NA | Fair | **Low** |
| Rodrigues et al. (2020), Portugal | Poor | Fair | Fair | NA | Fair | Poor | NA | NA | Fair | **Low** |
| Rodriques et al. (2021), Portugal | Poor | Fair | Fair | NA | Fair | Poor | NA | NA | Fair | **Low** |
| Sanders et al. (2016), USA | Poor | Fair | Good | NA | Poor | Poor | NA | NA | Good | **Low** |
| Sijtsma et al. (2015), The Netherlands | Fair | Fair | Fair | NA | Poor | Poor | NA | NA | Good | **Low** |
| Thompson et al. (2015), USA | Poor | Good | Good | NA | Poor | Good | NA | Fair | Good | **Low** |
| Thompson et al. (2018), USA | Poor | Fair | Good | NA | Fair | Poor | NA | NA | Fair | **Low** |
| Vaala and Hornik (2014), USA | Poor | Fair | Fair | NA | Poor | Poor | NA | NA | Good | **Low** |
| Waller et al. (2021), USA | Poor | Fair | Good | NA | Fair | Poor | NA | NA | Good | **Low** |
| Wang et al. (2020), China | Poor | Fair | Good | NA | Poor | Poor | NA | NA | Fair | **Low** |
| Wiseman et al. (2019), Australia | Poor | Fair | Poor | NA | Poor | Good | NA | NA | Good | **Low** |
| Xu et al. (2016), Australia | Fair | Good | Good | NA | Fair to poor | Good | NA | Poor | Fair | **Low** |

^1^ Items D and G were only applicable for intervention studies; ^2^ Scoring can differ when multiple correlates were examined, using different measurement instruments; ^3^ Item F2 was only applicable for longitudinal studies; Abbreviation: NA = Not applicable.
